# Cryogenic electron tomography by the numbers: Charting underexplored lineages in structural cell biology

**DOI:** 10.1073/pnas.2518350123

**Published:** 2026-02-18

**Authors:** T. Bertie Ansell, Louis Berrios, Kabir G. Peay, Peter D. Dahlberg

**Affiliations:** ^a^Department of Biology, Stanford University, Stanford, CA 94305; ^b^Division of Cryogenic electron microscopy and Bioimaging, SLAC National Accelerator Laboratory, Menlo Park, CA 94025; ^c^Department of Plant and Microbial Biology, University of Minnesota, St. Paul, MN 55108; ^d^Department of Earth System Science, Stanford University, Stanford, CA 94305

**Keywords:** cryogenic electron tomography, plunge-freezing, HPF, lamellae, biosphere

## Abstract

Imaging cells and their interactions across the whole biosphere with molecular-scale resolution is key for understanding structure–function relations. Cryogenic electron tomography (cryo-ET) is a powerful method for obtaining this critical information. However, cryo-ET studies are challenging and often limited to a small number of cell types per study. Here, we collate cryo-ET data from hundreds of cells and tissues across the biosphere to i) identify emerging methodological trends, ii) pinpoint strategies to reduce imaging time and costs, iii) quantitatively compare methods for cell freezing and sectioning, and iv) census cryo-ET species coverage across all domains of life. Comparing the fraction of cellular material within a single lamella across all domains of life reveals an order of magnitude difference between eukaryotes (1%) compared to bacteria (9%) and archaea (14%). We calculate the fraction of cellular material which can be imaged using distinct sectioning methods on multicellular communities and tissues—identifying serial lift-out as a powerful approach for obtaining more complete cellular depictions. Finally, we show that the biodiversity of current cryo-ET studies is 2 to 3 orders of magnitude lower than in sequence libraries and 4 to 5 lower than the total predicted on Earth. Our analyses reveal major evolutionary lineages which remain critically understudied and highlight where future cryo-ET research would be most impactful.

## Significance

Cryogenic electron tomography (cryo-ET) is a powerful method for imaging inside cells, but it is challenging and typically limited to a few cells per study. We consolidate and quantify data from hundreds of cellular cryo-ET studies across the biosphere. The methodological trends we identify will heighten biological insight from underexplored lineages of life on Earth.

Cryogenic electron tomography (cryo-ET) is emerging as the standout method for label-free, nonperturbative imaging of biological material with nanoscale resolution (1, 2). It is currently the only imaging modality, for instance, which routinely achieves resolutions of 2 to 4 nm while preserving cells in their native, hydrated states (3, 4). This powerful method has resolved key subcellular processes in exquisite detail including, but not limited to, i) in situ structures of bacterial flagella (5) and ii) injection complexes (6) which could not be easily purified, iii) visualization of cytoskeletal arrangements that enable intracellular trafficking processes (7, 8), iv) viral capsid structures and infection cycles (9), v) the native arrangements and heterogeneity of protein complexes such as ribosomes (10) and vi) partitioning of phase-separated regions (11). The resolution provided by cryo-ET therefore has and continues to yield unparalleled knowledge of biological structures and novel insights into how life functions (12).

Nonetheless, the cryo-ET pipeline for data collection—not including analysis—is often cumbersome, with typical timelines of ~1 to 2 wk for culturing cells, freezing grids, sectioning samples, and obtaining tomograms. Furthermore, each stage requires highly skilled manual intervention to transfer samples between the different instruments and manipulate them in situ. For context, we estimate the total cost of cryo-ET to be ~US$3 to 5 k for a single imaging round, factoring in grid costs, sample preparation, and imaging using bespoke equipment or user fees. These high user costs reflect the steep price of instrumentation purchase, maintenance, and expert staff, leading to facility operational budgets on the order of millions US$ per year (13). This is 2 to 4 orders of magnitude greater than cellular light-microscopy (14), and is a substantial barrier to cryo-ET research. Resultantly, these constraints tend to limit studies to only one (5) or a small subset of cell types (15). The field would certainly benefit from efforts that reduce time and costs and enhance cryo-ET throughput.

One potential solution to these problems is to clarify and consolidate reproducible methods needed to scale cryo-ET applications for imaging across our vast biosphere and identify critical transition points between approaches. These analyses would help 1) identify methodological trends to speed up experimental proceedings, 2) reduce time and labor costs, 3) identify potential paradigm-shifting techniques for moving structural analyses toward more complete depictions of whole cells/organisms, 4) census species coverage within the literature and 5) identify underrepresented areas of life to target in future cryo-ET studies. While there are several reviews which summarize the advances and limitations of cryo-ET (1, 16, 17), we are aware of no reports which systematically evaluate biodiversity and methodology used across cellular cryo-ET studies and which identify factors shaping methodological choice across all domains of life.

To achieve these aims, we first elaborate on technicalities of the cryo-ET pipeline necessary for preparation of cells or tissues. Cells must be cultured and frozen on electron microscopy (EM) grids or in planchettes by one of two processes: i) rapid plunging into liquid ethane under cryogenic temperatures or ii) contact with liquid nitrogen at ~2,100 bar within a high-pressure freezer (HPF), which depresses the sample freezing point and slows ice crystal growth (18–20). This results in a layer of vitrified (amorphously frozen) ice typically 1 to 10 μm (plunge freezing) or 10 to 200 μm thick (for HPF). These cryogenic approaches typically lead to reduced cellular artifacts like membrane perturbation compared to resin embedding or freeze-substituted samples. For imaging with the transmission electron microscope (TEM) samples must be <0.3 to 0.5 μm thick else inelastic scattering of electrons by the cellular material limits the resolution obtainable (21). Hence, thicker samples must be thinned, typically using a focused ion beam (FIB) of accelerated gallium or plasma ions (xenon, argon, oxygen) (22, 23). The FIB beam ablates cellular material with nanometer-scale precision above and below a thin slab known as a lamella. If the sample is extremely thick micromanipulation techniques may precede FIB milling of lamella, whereby a larger chunk of material is excised using the FIB and deposited on another grid using a needle. Excision of a single block of material is called cryo-FIB lift-out (24). In serial lift-out 3 to 4 μm subsegments of this block are excised and redeposited for independent sectioning (25, 26). An alternative sectioning method involves using an ultramicrotome to precisely cut cellular material using a diamond knife (27). Finally, once a ~0.2 μm lamella has been produced, the grid is transferred to the TEM where it is sequentially tilted and imaged to generate a cryo-ET tomogram. Subsequent computational processing and imaging segmentation steps are performed; however, these are not the focus of this study and are detailed elsewhere (28–31). Hence, this overview spotlights the considerable expertise necessary for production of a small number of lamellae, which typically account for a tiny subset of the total cellular material. Thus, efforts to consolidate studies and identify trends are essential.

In this study, we consolidated cryo-ET data from hundreds of cell or tissue types across bacterial, archaeal, and eukaryotic domains of life. One key goal of this analysis was to identify cellular traits which shape transitions between methodological approaches. We also assess which methods move the field closer to imaging whole cells/tissues—a vital methodological advancement needed for tracing structural architectures across biochemical and spatial gradients. Additionally, we quantitatively map the fraction of cellular material captured within lamellae for distinct cell types and preparation methods, comparing time and imaging efficiencies to maximize cellular insight. This comparative statistical analysis of imaged material within lamellae across the biosphere therefore aimed to strengthen prior estimates of 0.5 to 4% (32) and direct future efforts toward increased cell coverage via emerging methods. Finally, we compared the biodiversity within cryo-ET studies to both the sequence diversity in publicly available databases and the total predicted biodiversity on Earth. Our findings expose major evolutionary lineages that remain poorly studied and ripe for cryo-ET exploration.

## Methods

### Literature Search.

Three independent literature searches were performed using the “Web of Science” database with the following terms: i) “cryogenic electron tomography,” ii) “electron cryotomography,” and iii) “cryoelectron tomography” (www.webofscience.com). Entries were filtered using the “Cell Biology” category to eliminate papers from materials or the physical sciences before amalgamating entries across the three search terms. The literature search was initially performed in August 2024 and updated in November 2025 during review. A total of 730 publications from 1984 to the end of 2024 were identified and filtered further by manual inspection using the following criteria. Results were refined by excluding studies which 1) used resin embedding for sample preparation, 2) did not use intact cells (e.g., visualized proteins, fibrils, cell walls, or virus particles), or 3) did not report sufficient methodological cryo-ET details to replicate the study and extract key parameters such as the freezing or sectioning approach. In addition, reviews, abstracts, and thesis dissertations were excluded, as were studies that only performed cryo-TEM. Papers reporting cryo-ET method development, which met these criteria, were included. The remaining 193 studies (beginning in 2003) were primary research articles which performed cryo-ET on cells. We subsequently supplemented entries using The Atlas of Bacterial and Archaeal Cell Structure by manually cataloguing articles associated with individual entries within the Jensen Lab cell atlas (33). This gave an additional 34 entries, bringing the total number of articles to 227.

For papers that reported cryo-ET on multiple cells/tissues (54 studies), repeat entries were created for each cell/tissue type. Hence, a representative sample of 366 cells or tissues are included within this meta-analysis (*SI Appendix*, Table S1). Bacterial and archaeal cells are listed according to genus and eukaryotic cells are listed according to cell line or genus (if applicable). There were 164 cryo-ET imaging events of bacterial, 14 archaeal, and 188 eukaryotic cells each. The choice of search term resulted in differing distributions of bacterial, eukaryotic, and archaeal cells (*SI Appendix*, Table S2), reflecting field-specific preferences. Amalgamating over three searches helped ensure a varied and reflective sampling of the cryo-ET literature.

Each entry was manually inspected and the methods used for freezing and sectioning of cells/tissues were recorded in *SI Appendix*, Table S1, including chemical details of cryoprotectants used for a subset of cells (39 entries). Throughout, we use sectioning to refer to all processes of cellular thinning which lead to lamellae production, including use of the FIB. This dataset is not an exhaustive list of all cryo-ET studies performed to date since this is not feasible and manual inspection was essential to extract methodological details. However, it is a representative sample of cryo-ET studies, which intends to accurately capture the taxonomic diversity of cells studied.

### Cell/Tissue Size Estimates.

Cell/tissue sizes (lengths, widths/thicknesses) were mostly derived from the literature. If a range of lengths/widths was given the mean value was used. For entries taken from The Atlas of Bacterial and Archaeal Cell Structure (33) cellular dimensions were measured directly from images in the database if the entire cell was visible, else derived from the literature (*SI Appendix*, Table S1). Each cell was described as either a capsule, sphere, square, rectangle, cone or rectangular pyramid, and the shape dimensions were recorded. For example, the length and width/thickness were reported for capsule, spherical, square and conical cells. For rectangular and rectangular pyramid shaped cells an additional length dimension was recorded.

### Analysis of Imaged versus Total Cellular Fractions.

The percentage of a cell imaged within a lamella (P) was defined using Eq. **1** where $N_{l}$ corresponds to the maximum number of lamellae that could be produced by following a standard sectioning workflow once for a given cell/tissue. For FIB-SEM and cryo-FIB lift-out, a single lamella ($N_{l}=1$) is produced for each round of sectioning, whereas for serial lift-out and the ultramicrotome the cell length (L) was divided by the minimum theoretical separation distance between lamellae (~10 ⨯ 10 ⨯ 0.18 μm^3^), assuming a lamellae width (l) of 0.18 μm (34). This separation distance was therefore ~4 μm for serial lift-out (25, 26) (although sections <4 μm are also possible) or 0.18 μm (i.e. no separation between lamellae) with the ultramicrotome. Application of this equation to four commonly studied cells/species (**Escherichia coli*, Chlamydomonas reinhardtii*, HeLa, and *Caenorhabditis elegans*) for each sectioning approach (none, FIB-SEM, cryo-FIB lift-out, serial lift-out, or ultramicrotome) is demonstrated in *SI Appendix*, Fig. S1.

[1] $$P=\frac{l*N_{l}}{L}*100.$$

We also tried estimating the fraction of a cell contained within a lamella by comparing lamella volumes to total cellular volumes. However, these estimates were less robust for comparative analyses across domains because lamellae volumes were typically larger than total bacterial and archaeal cell volumes.

### Time and Yield Analysis of Combined Freezing and Sectioning Approaches.

Three combinatorial freezing and sectioning approaches (“Plunge + FIB-SEM,” “HPF + Waffle,” and “HPF + Serial lift-out”) were selected to compare differences in the preparation time, imaging efficiencies, and cellular insight across different sample preparation strategies for cryo-ET. Sample preparation strategies for four small, evolutionarily diverse, and commonly studied cell types were compared (Bacteria: *E. coli* biofilm, Fungi: *Saccharomyces cerevisiae*, Protista: *C. reinhardtii*, and Animalia: HeLa). The maximal number of cells per lamella ($N_{c}$) was calculated by dividing the lamellae cross-sectional area ($A_{l}$) by the cellular area ($A_{c}$) (Eq. **2**). Lamellae cross-sectional areas were taken from the literature (25, 26, 35, 36): i) “Plunge + FIB-SEM” 10 ⨯ 10 = 100 μm^2^ for all eukaryotic cells or 5 ⨯ 10 = 50 μm^2^ for the bacterial biofilm, ii) “HPF + Waffle” 20 ⨯ 20 = 400 μm^2^ and iii) “HPF + Serial lift-out” 10 ⨯ 25 = 250 μm^2^. Cellular cross-sectional areas were calculated using the lengths listed in *SI Appendix*, Table S1 and approximation to a sphere: i) *E. coli* 2 μm^2^, ii) *S. cerevisiae* 19.6 μm^2^, iii) *C. reinhardtii* 50.3 μm^2^, and iv) HeLa 227.0 μm^2^. While these data are empirical estimates, they align with the predicted number of cells per lamellae reported experimentally (35).

[2] $$N_{c}=\frac{A_{l}}{A_{c}}.$$

The preparation time for 100 cells ($T_{100c}$) was calculated using Eq. **3** where $T_{l}$ is the time taken to mill a single lamella including any associated trenching or lift-out steps. $T_{l}$ was taken to be 60 min for conventional FIB-SEM, 150 min for Waffle and 75 min for serial lift-out (25, 26, 36). An imaging area of 12.6 μm^2^ ($A=\pi r^{2}=4\pi$) was used to calculate the maximal number of tomograms per lamellae based on a typical illumination area of a 1.8 ⨯ 1.8 = 3.24 μm^2^ tomogram with a 4.4 Å pixel size.

[3] $$T_{100c}=T_{l}\frac{100}{N_{c}}.$$

### Comparison of Cryo-ET Entries with Global Sequencing Abundance.

The number of recorded prokaryotic genera was obtained from the PATRIC database (version 3.49.1, accessed December 2024) based upon the number of entries with rRNA sequencing data (https://www.bv-brc.org/view/Bacteria/2) (37), and the total predicted genera on Earth are reported in the SILVA database (38, 39) (version 138.2, accessed December 2024). The number of recorded eukaryotic genera was obtained from rRNA entries within EUKARYOME (40) (version 1.9.4, accessed April 2025) while the total predicted genera were obtained from the Interim Register of Marine and Nonmarine Genera (IRMNG) for extant and accepted eukaryotic entries (41) (accessed April 2025). The predicted number of eukaryotic species by kingdom was derived from reported taxonomic estimations (42). The number of human cell line entries was derived from those listed in the Human Protein Atlas based on RNA sequencing (https://www.proteinatlas.org/humanproteome/cell+line/data#cell_lines) (43, 44). Estimates and database entries across kingdoms are unlikely to be numerically accurate representations of species diversity but are necessary approximations for proportional abundances on Earth and are useful for comparisons.

### Data Analysis and Summary Reporting.

For statistical analyses of the amount of cellular material within lamellae a Kruskal–Wallis test was performed (*p* = 4 ⨯ 10^−45^) followed by a pairwise Dunn’s tests between domains (Bact-Euk: *P* = 1 ⨯ 10^−41^, Bact.-Arch: *P* = 3 ⨯ 10^−1^, Euk-Arch: *P* = 3 ⨯ 10^−11^) using the SciPy and Scikit-posthoc python packages (45, 46). The Kruskal–Wallis test was selected because data were not normally distributed and there were different sample sizes across domains. Analysis of data was conducted in python and both the code and associated datasets are deposited on Zenodo (10.5281/zenodo.15831544). Details of cell studies and publication are provided with the *SI Appendix*.

## Results

### The Emerging Cellular Cryo-ET Revolution.

To evaluate the current landscape of cellular cryo-ET research, we conducted a meta-analysis of 366 cell or tissue types. Using data pooled from 227 primary research articles released between 2003 and 2024, we consolidated studies of 164 bacterial, 188 eukaryotic, and 14 archaeal cells (*SI Appendix*, Table S1) to address outstanding information gaps within the literature. Notably to:

• Map methods for freezing and sectioning across a range of cell types.
• Identify factors which shape transition points between methodological approaches.
• Quantitatively assess the fraction of cellular material captured within lamellae.
• Highlight powerful but underutilized methodological approaches.
• Identify underexplored kingdoms of life to expedite future cryo-ET research.

**Fig. 1.**
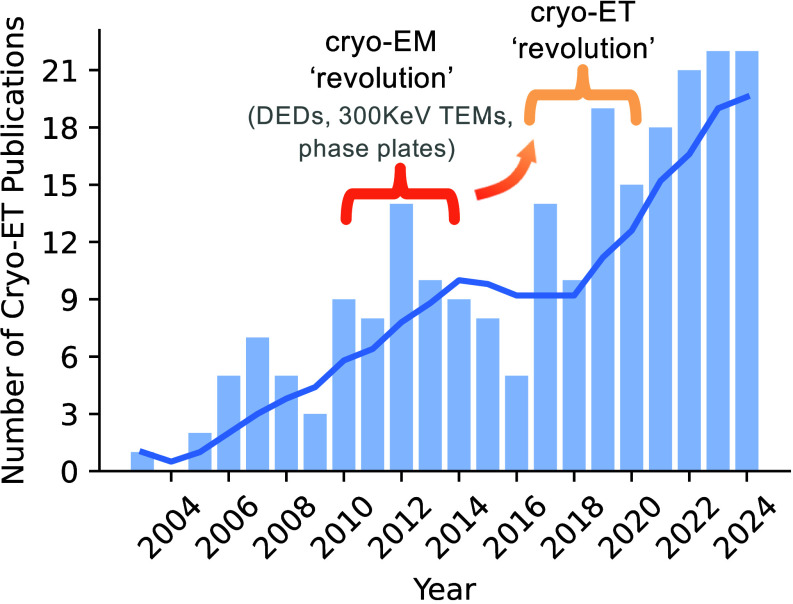
The emergence of a cellular cryo-ET revolution. Summary of the number of filtered publications by year included within this analysis until the end of 2024. The solid line indicates the rolling mean over 5 y. Articles were pooled between 1984 and 2024 and filtered manually based on specified search criteria (*methods*). Thus, the remaining filtered publications accounted for the years 2003 to 2024.

The cryo-EM “resolution revolution” can be attributed to the years starting ~2012 due to the innovation and commercialization of direct electron detectors (2004, 2008) (47), Volta phase plates (2014) (48), 300 KeV electron microscopes, and advances in computational image processing (1, 29, 31, 49). Compared to the so-called cryo-EM “resolution revolution,” the increase in cellular cryo-ET studies was shifted by approximately 6 y, likely due to the added complexity of in situ imaging (Fig. 1 and *SI Appendix*, Fig. S2). While milling methods existed prior to this period, the commercialization of equipment contributed to cryo-ET uptake during this time. Likewise, image analysis and machine learning approaches to in situ high-resolution structure determination are developing rapidly (50, 51). Hence, this systematic analysis of cellular cryo-ET studies is aptly timed to assess the current state of the field and where future efforts would be most impactful.

### Cell Size Dictates Choice of Freezing and Sectioning Approach.

**Fig. 2.**
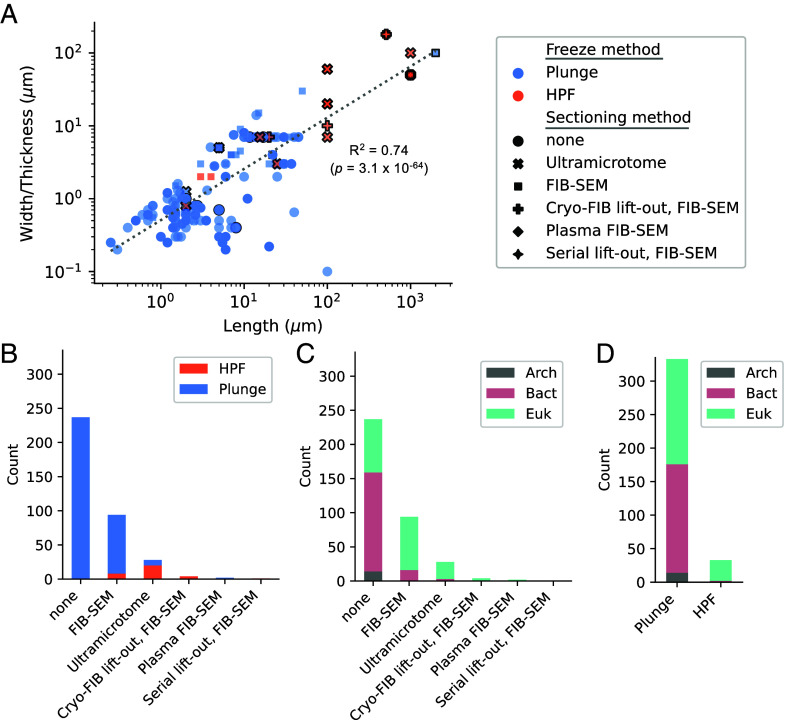
Cryo-ET methods used to freeze and section cellular material. (*A*) Scatter plot of the cell/tissue length versus width/thickness for the 366 cells within this study, colored by freezing method (plunge freezing: blue, HPF: orange). Scatter plot marker types indicate the sectioning method used (●: none, x: ultramicrotome, ■: FIB-SEM, ♦: plasma-FIB, ✚: cryo-FIB lift-out, FIB-SEM, ✦: serial lift-out, FIB-SEM). A solid marker outline indicates imaging events in which cryoprotectant was used. (*B*) Distribution of cryo-ET sectioning methods colored by freezing type or (*C*) domain of life. (*D*) Distribution of cryo-ET freezing methods colored by domain of life.

First, we sought to quantify the extent to which specific cellular traits, such as size or cellular composition, drive switching between cryo-ET freezing methods (52). We plotted the length versus width of cells/tissues and colored these based on whether plunge freezing or (Fig. 2*A*, blue) or high-pressure freezing (HPF) (Fig. 2*A*, red) was used for vitrification. Entries were linearly distributed along cellular dimensional scales (R^2^ = 0.74, *P* = 3.1 ⨯ 10^−64^) and ranged from 0.1 to 0.2 μm at the smallest scales to 100 to 2,000 μm for multicellular material. We found that a transition between plunge freezing and HPF typically occurred when one cellular dimension surpassed 100 μm, yet there were many studies above this cut-off which employed plunge freezing. The maximal tissue dimensions vitrified with plunge freezing were for human brain tissue (100 μm ⨯ 2,000 μm) and *C. elegans* embryos (30 μm ⨯ 50 μm), both in the presence of cryoprotectant (25, 53). Hence, cell *size* is the fundamental driver for choice of freezing approach and appears to have greater weighting than the cellular *composition*. While this may seem obvious to experienced cryo-ET scientists, these data will help streamline new users toward viable methodological combinations for specific cell types. One noteworthy subtlety was that cryoprotectant use dominated in samples with dimensions >200 μm, effectively extending the upper limit considered viable for vitrification (Fig. 2*A*, black outline).

We next compared sectioning and freezing methods across studies to identify potential correlations between cell types and methods. Sectioning methods included intact cells (i.e., no sectioning), FIB-SEM, plasma-FIB, ultramicrotome, cryo-FIB lift-out, serial lift-out or a combination, while freezing methods included either plunge freezing or HPF. In most cases cells were not sectioned (65% of total, Fig. 2*B*), vitrified by plunge freezing, and bacterial (61% of nonsectioned entries) (Fig. 2 *B* and *C*). The next most common approaches were FIB-SEM (26%) followed by the ultramicrotome (8%) and cryo-FIB lift-out (1%) (Fig. 2*B*). Of these, eukaryotic cells dominated usage (85% of sectioned cells), and HPF was used for 9% of FIB-SEM and 71% of ultramicrotome sectioned cells (Fig. 2 *B* –*D*). Plasma-FIB data are sparsely represented in this meta-analysis as it has only recently begun to be used for biological material. Finally, there were five studies which used cryo-FIB lift-out or serial lift-out followed by the FIB-SEM to study extremely large eukaryotic tissues: *D. melanogaster* eggs and *C. elegans*, respectively, which were frozen by HPF (24, 25) (Fig. 2 *B–D*).

### Quantification of the Fraction of Cellular Material Captured within Lamellae across the Biosphere.

**Fig. 3.**
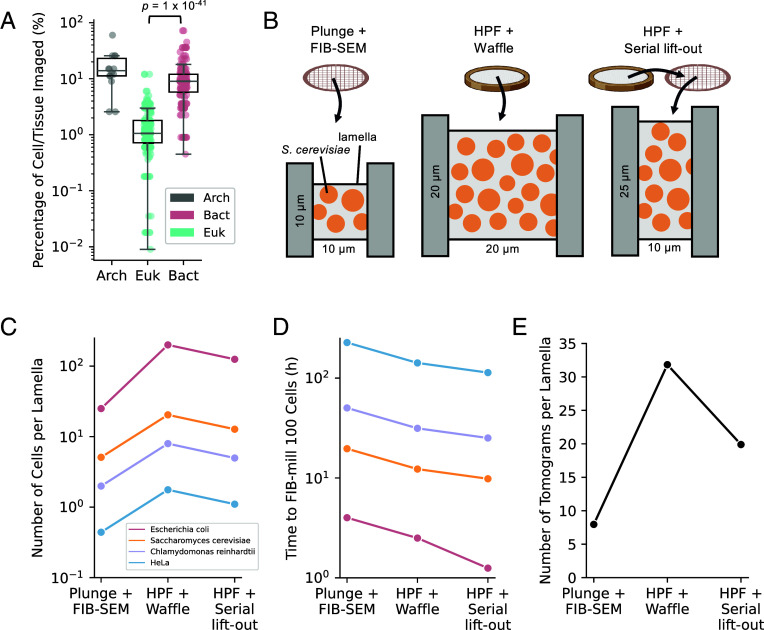
The fraction of cellular material captured within lamellae and systematic comparison of methods to optimize cellular insights by cryo-ET. (*A*) The percentage of cellular material captured within a lamella for each cell/tissue type across bacterial (red), eukaryotic (teal), and archaeal (gray) domains. The percentage imaged was defined as in Eq. **1** whereby a standard lamellae width (l=0.18μm) was divided by the maximal dimension from Fig. 2*A* for each cell/tissue. Significance was tested using a Kruskal–Wallis test (*P* = 4 ⨯ 10^−45^) followed by a pairwise Dunn’s test between domains (Bact-Euk: *P* = 1 ⨯ 10^−41^, Bact.-Arch: *P* = 3 ⨯ 10^−1^, Euk-Arch: *P* = 3 ⨯ 10^−11^). (*B*) Schematic of three different preparation methods for small, commonly studied cells by i) plunge freezing + FIB-SEM, ii) HPF + the Waffle method, and iii) HPF + serial lift-out. Typical lamellae sizes are marked and numerous cells within lamellae are depicted. Comparison of (*C*) the number of cells within a single lamella (Eq. **2**) and (*D*) the time taken to mill 100 cells (across multiple lamellae) via each freezing and sectioning combination (Eq. **3**) for four cell types (*E. coli* biofilms: red, *S. cerevisiae*: orange, *C. reinhardtii*: purple, and HeLa: blue). (*E*) Theoretical number of tomograms which can be obtained per lamellae for each approach assuming typical illumination areas corresponding to 4.4 Å/pixel.

Cryo-ET lamellae represent only a tiny fraction of total cellular material, estimated at 0.5 to 4% for a typical mammalian cell (32). To provide a more granular assessment, we quantified lamellae fractions across all entries to ultimately assess how much cellular material is captured for individual cell types and organisms. We first calculated the fraction of cell/tissue material imaged assuming one lamella had been produced, with a typical lamellae width of 0.18 μm for FIB-milled samples (34). We then divided this by the maximal length dimension from Fig. 2*A*. For bacterial and archaeal cells, the median percentage of cellular material imaged was 9% and 14%, respectively, whereas for eukaryotes, this was significantly lower (1%) (Fig. 3*A*). In extreme cases, only 0.009 to 0.7% of eukaryotic cellular material were represented in lamellae. These data align with estimates of lamellae fractions within the literature (32, 54) but further quantify and contextualize these fractions across multiple domains of life and for individual cell types (*SI Appendix*, Table S1). This quantification shows that while lamellae from bacterial and archaeal cells capture an order of magnitude more cellular volume than those from eukaryotes, they still represent only a small fraction of the whole cell volume. This also spotlights the need for complementary and/or correlated contextualizing experiments (11, 55) to build statistically robust structure–function models (56).

Given these small fractions, we explored the potential of combined freezing and sectioning workflows to capture more cellular insight while minimizing preparation times. We calculated the theoretical maximum number of cells per lamella for four commonly studied and evolutionarily diverse organisms/cells (*E. coli* biofilms*, *S. cerevisiae*, C. reinhardtii,* and HeLa) assuming a standard workflow was followed to completion for each of i) plunge freezing and on-grid FIB-SEM, ii) HPF and the Waffle milling approach, or iii) HPF and serial lift-out (Fig. 3 *B* and *C*) (25, 26, 36). The number of cells per lamella was roughly 25 (*E. coli* biofilms), 5 (*S. cerevisiae*), 2 (*C. reinhardtii*), or 0.5 (HeLa) for plunge freezing combined with FIB-SEM, in line with experimental values (35). The number of cells per lamella increased 3 to 4 x for the HPF approaches due to the larger lamellae areas. While HPF combined with the Waffle method gave the greatest number of cells per lamellae, the time associated with milling and trenching was longer (~150 min per lamellae) than for serial lift-out (~75 min). Hence, when we calculated the time taken to expose 100 cells by each hybrid approach across multiple lamellae, the net time decreased across approaches and HPF plus serial lift-out emerged as the most time efficient method to maximize cellular insight (Fig. 3*D*). Next, we calculated the number of tomograms which could be produced per lamella, assuming an exposure area typical of a 4.4 Å/pixel tomogram (Fig. 3*E*). HPF combined with the Waffle method gave ~32 tomograms, followed by ~20 tomograms from HPF plus serial lift-out and ~8 from plunge freezing and FIB-SEM. Systematic comparison of the maximal number of lamellae and fraction of material imaged for sectioning larger tissues also reveals serial lift-out as a powerful method, particularly for pristinely preserving native architectures which can be perturbed by use of an ultramicrotome (27) (*SI Appendix*, Fig. S1). Hence, combining HPF with either the Waffle method or serial-lift out offers a substantial advantage in maximizing cellular insights and streamlining data acquisition compared with conventional plunge freezing with on-grid FIB-milling.

### Extensive and Diverse Clades of Life Remain Unexplored by Cryo-ET.

**Fig. 4.**
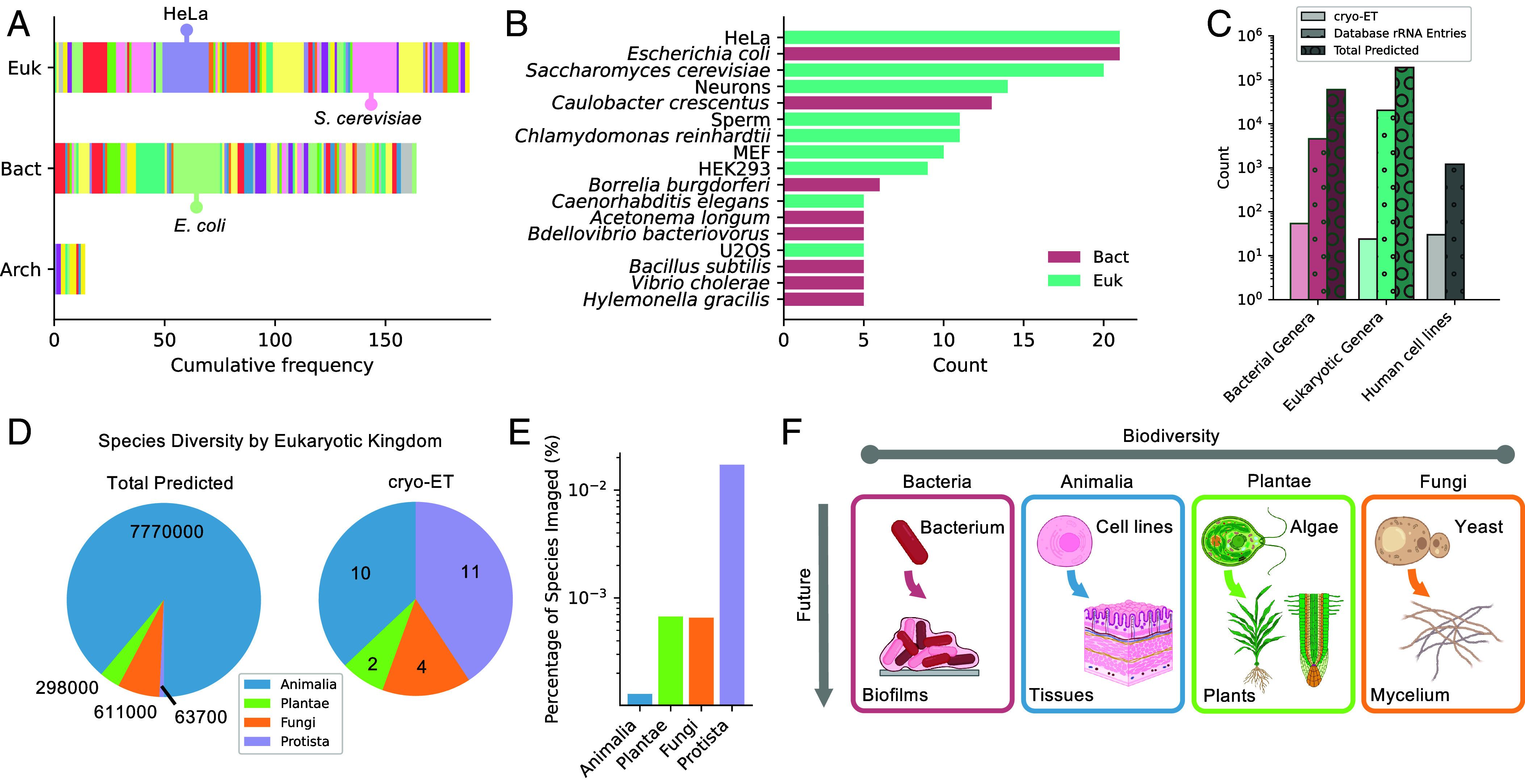
The biodiversity of cryo-ET compared to globally. (*A*) Cumulative frequency of cell/tissue types for all 366 eukaryotic, bacterial, and archaeal cryo-ET imaging events colored by individual species or cell lines. Entries are in alphabetical order and correspond to those in *SI Appendix*, Table S1. The top three cell types are marked. (*B*) Top species or cell lines studied by cryo-ET. (*C*) Comparison of the number of distinct bacterial genera (red), eukaryotic genera (teal), and human cell lines (gray) within cryo-ET imaging (solid bars), rRNA databases (dots) and the total predicted on Earth (circles). The number of sequenced prokaryotic and eukaryotic genera was obtained from the PATRIC and EUKARYOME databases, respectively (37, 40). Estimates of total richness were obtained from the SILVA and IRMNG registers (38, 41). The number of total human cell lines was derived from refs. 43, 44. (*D*) Comparison of the number of unique eukaryotic species predicted globally (42) versus those imaged by cryo-ET (excluding cell lines) across animalia, plantae, fungi, and protista kingdoms. (*E*) Percentage of unique eukaryotic species in cryo-ET publications versus total predictions for each kingdom. (*F*) Summary of underexplored multicellular architectures and clades of life to guide future cryo-ET studies.

One goal of this analysis was to assess the explored and unexplored search space of cellular diversity in cryo-ET studies. First, to assess which cells/tissues are frequently studied via cryo-ET, we calculated the number of entries for each cell/tissue type across domains of life (Fig. 4*A* and *SI Appendix*, Table S1). There were 70 species of bacteria represented within our analyses, of which easily culturable, genetically tractable and thin species such as *E. coli*, *C. crescentus* and *B. burgdorferi* were the most frequently represented (Fig. 4*B*). For eukaryotes, 62 species or cell lines were represented, and the most frequent occurrences were for single celled eukaryotes such as *S. cerevisiae* and neurons, or widely used cell lines (HeLa, HEK293) (Fig. 4 *A* and *B*). In almost all cases that did not use cell lines, studies were restricted to model organisms (*S. cerevisiae, C. reinhardtii, D. melanogaster, C. elegans*). For archaea, there were 12 species represented, but studies were sparse and did not account for any of the most frequently studied model organisms (Fig. 4*B*).

Next, we compared the number of genera within our set of representative cryo-ET studies to those annotated within rRNA databases and to the total number of predicted genera on Earth (Fig. 4*C*). The goal of this analysis was to compare cryo-ET to well established sequencing methods by assessing how much of the biosphere is currently represented, and where cryo-ET has the potential to be as it develops from methodological infancy. For bacteria and eukaryotes, the number of genera imaged was 2 to 3 orders of magnitude lower than those in sequence libraries (37, 40), and 4 to 5 orders of magnitude lower than the predicted planetary abundances (38, 39, 41). We performed a parallel comparison for distinct human cell lines studied by cryo-ET and within rRNA databases, which also differed by three orders of magnitude (43, 44) (Fig. 4*C*). Moreover, the number of human cell lines represented was almost identical to the total diversity of eukaryotic genera studied by cryo-ET. In summary, the current biodiversity of cryo-ET studies is low and there is ample scope for expanding species representation as the method expands into unexplored areas of life.

To dissect whether eukaryotic studies (excluding cell lines) proportionally reflected the distribution of life on Earth, we compared the total predicted number of species in the kingdom Eukarya to the number of distinct species imaged by cryo-ET (Fig. 4*D*). Animalia are the most diverse eukaryotic kingdom, predicted to account for 89% of total species richness (most of which are arthropods) (42) and yet accounted for only 37% of cryo-ET studies by kingdom. In contrast, plantae, fungi, and protists are predicted to account for 3%, 7%, and 1%, respectively (42) but were proportionally much higher in cryo-ET studies (plantae: 7%, fungi: 15%, protista: 41%). Hence, the species richness of eukaryotic kingdoms in cryo-ET studies does not mirror the abundances of life on Earth and accounts for only a tiny proportion of the totals predicted (0.0001 to 0.02%) (Fig. 4*E*).

For animals, cryo-ET studies of human, mice, and macaque tissues have been conducted in addition to the model organisms, *C. elegans* and *D. melanogaster* (53, 56–58). For fungi, yeasts have been studied in their nonfilamentous forms (*S. cerevisiae*), in addition to species within the microsporidium division (*Anncaliia algerae, Encephalitozoon hellem*) (23, 36, 59). Plant studies were restricted to unicellular green algae (*Ostreococcus tauri, C. reinhardtii*) or spinach leaf (60–63) while protists were parasites (*Plasmodium berghei, Toxoplasma gondii, Trypanosoma brucei*) (8, 64–66) or oceanic coccolithophores (*Gephyrocapsa huxleyi*) (15). Hence, outside of the animal kingdom, eukaryotic cryo-ET studies have only been performed on unicellular organisms, illuminating a path toward future cryo-ET studies on diverse multicellular organisms which remain architecturally enigmatic.

## Discussion

### Current and Future Trajectories of Cryo-ET Methodology.

Our assessment of cryo-ET data provides an integrated consortium of freezing and sectioning approaches to cellular material across all domains of life. This foundation has primed researchers to explore a wealth of cellular anatomies as the adoption of cryo-ET continues to gain momentum (Fig. 1). Hereafter, we outline a series of methodological principles to guide future cryo-ET studies and accelerate in situ cellular discovery.

We aggregated the freezing and sectioning approaches used for cells/tissue across all domains of life (Fig. 2). These data are intended to benefit the community by reducing the time and cost associated with optimization of samples, enhance the accessibility of cryo-ET for new users, and pinpoint appropriate pairwise combinations of vitrification and sectioning methods. Since vitrification is a key bottleneck for cryo-ET, we provide a comprehensive breakdown of preparation conditions for individual cells/tissues to compare or expand upon established studies or facilitate automated preparation (*SI Appendix*, Table S1). Our analyses show that bacterial and archaeal cells can be routinely plunge frozen and require minimal to no sectioning, provided cells are sparsely distributed on the grid and are hydrated (Fig. 2 *A–D*). For bacterial cells thicker than 300 nm, FIB-SEM sectioning is the primary methodological approach. Increased cell density or width of the hydrated layer, however, may be required to prevent grid buckling during milling. For unicellular eukaryotic cells, the most frequent pipeline was plunge freezing followed by gallium or plasma FIB milling (Fig. 2 *A–D*). Once cell/tissue dimensions surpass ~10 to 100 μm, it is usually appropriate to switch from plunge freezing to HPF and the vitrification threshold can be extended beyond 200 μm by cryoprotectant use (Fig. 2*A*). Further assessments are required to systematically compare how HPF preservation, particularly for plant and animal tissues, is affected by choice of cryoprotectant.

Expanding upon these foundational principles, we highlight several powerful approaches which push the boundaries of current cryo-ET methodology. First, plasma-FIB use was underrepresented in our analysis because the methodology is still emerging. We anticipate plasma-FIB uptake to increase, especially for eukaryotic cells, as material can be sectioned quickly and may have reduced sample damage compared to the traditional gallium FIB depending on choice of plasma (22, 67). Second, a specialized use of HPF is to freeze smaller cells such as bacterial layers via the “Waffle method” (36) (Fig. 3). This would be highly valuable for studying multiple cells within a single lamella *en mass* and understanding how cells are situated within their surrounding environment, e.g., in biofilms where cell–cell and cell–extracellular matrix interactions are critical (68). This could also assist immune or signaling insights such as antibody conjugation to specific extracellular targets or in vesicle release. Next, our assessment of the potential for different sectioning approaches to image greater proportions of cells than the 1 to 10% typically imaged (Fig. 3*A*) identified HPF plus serial lift-out as high-potential method (24–26). This approach had higher numbers of cells per lamella with reduced preparation times compared to conventional FIB-SEM approaches on plunge-frozen grids (Fig. 3 *B–E*) but was only sparsely represented within our meta-analyses (25). This will become important for using cryo-ET to understand how cellular heterogeneity, e.g., via rare subpopulations of cells, confers fitness advantages to the community (69) or how architectures emerge in time-dependent processes like biofilm formation or quorum sensing.

Since 2024, serial lift-out has been successfully used to obtain vitrified plant protonemata from *Physcomitrium patens* with an adapted single-sided needle attachment for faster manipulation (54). These studies are a substantial advancement for studying i) plant tissues and ii) all multicellular life because they highlight the increased biological insight that is obtained by tracing structural architectures along the same organism. For example, serial lift-out applied to *C. elegans* revealed packing arrangements of actin–myosin bundles along larvae which could not be inferred from individual lamellae (25). There are other challenges to preparing specific cells for vitrification, including delicate filamentous structures or exoskeletons which do not FIB-mill easily, high salt concentrations or sample fractures due to air pockets such as vacuoles. For some samples, alternative, cheaper ultrastructural imaging methods like expansion microscopy, light-sheet microscopy or volume-EM may be more tractable approaches for understanding cellular organization.

Finally, in preparing this manuscript, it became apparent that there is a growing need for standardized data reporting such as within imaging databases (CZII, EMPIAR) (70, 71) which would assist automation and AI feature recognition. We are on the precipice of being able to visualize structures across length-scales to trace bioanatomical features across whole organisms. For processes where chemical and environmental gradients are important for structural emergence, such as in developmental biology, molecular scale resolution across these axes will be powerful.

### What Contributes to Existing Biases in Cryo-ET Research?

After identifying methodology to better capture structural landscapes, the question remains which organisms to target for maximizing biological discovery. Across kingdoms, the biodiversity captured by cryo-ET is 2 to 5 orders of magnitude lower than those in sequence databases or on Earth (Fig. 4). Cryo-ET is young, challenging, and limited in scale-up compared to other imaging methods, with each factor contributing to undersampling of biodiversity. Understandably, most cryo-ET studies have focused on unicellular life or cell lines to meet requirements for thin samples, liquid culturing or where there is clear, preexisting evidence from other studies for the necessity of nanoscale depictions (Fig. 4 *A* and *B*). The species biases we observe reflect patterns common across bioimaging and stem in part from long-standing trends in cell biology toward model systems with biomedical applications. Funding for high-cost techniques like cryo-ET is often directed toward projects supported by prior biochemical or physiological evidence and to institutions with established infrastructure – factors that disproportionately favor the global North.

While commonly studied organisms are useful for method development and benchmarking (4, 55, 57), we must be cautious not to create too much redundancy or repetition bias within the literature (Fig. 4*B* and *SI Appendix*, Table S2). Shifting these biases will require a renewed focus on discovery-driven research and a broader exploration of nonpathogenic and environmentally relevant organisms across diverse ecological contexts. For example, studying how architectures differ between species can inform functional and evolutionary trends. This is evidenced by recent cryo-ET structures of pyrenoid compartments from the diatoms *Phaeodactylum tricornutum* and *Thalassiosira pseudonana* which have distinct thylakoid sheets and rubisco partitioning that may alter gaseous diffusion compared to in well studied *C. reinhardtii* pyrenoids (72). Further work is also needed to assess whether the immortalized nature of eukaryotic cell lines results in architectures which may not be reflective of healthy cells or tissues (73). Hence, expanding beyond model organisms is functionally necessary for revealing untapped cellular information by cryo-ET (Fig. 4*C*).

### Chartering Underexplored Lineages in Structural Cell Biology.

We identified underexplored clades of life where future cryo-ET studies would be welcomed and feasible (Fig. 4*F*). For eukaryotes, animalia tissues, embryophyte plantae and filamentous fungi are ripe for exploration. Recent, pioneering studies of human and mouse brain tissue revealed the arrangement of fibrilla plaques implicated in Alzheimers (53, 58) and the in situ location of synaptic glutamate receptors (74). Importantly, these explorations of animal tissues were only made possible by advances in sample manipulation with cryogenic needles, as discussed above. One critical dimension that remains underexplored is imaging multispecies interactions in disease (65) and ecological contexts (75). For example, a study of the *Plasmodium falciparum* infection cycle in human erythrocytes showed altered ribosomal abundances when parasites were first treated with drugs (76). This highlights how cryo-ET can be used to assess how multicellular landscapes are shifted by pharmaceutical or environmental intervention. Additional areas where structural insights would be welcomed include i) comparing domesticated lab strains with those isolated from environmental or disease contexts such as for *Pseudomonas* strains, ii) studying aquatic microbes in low-salt ecological contexts including fresh water fungi like chytrids, iii) examining structural adaptations that enable microbial survival at extremes e.g., arctic conditions, hydrothermal vents, ammonified aquifers, iv) evaluating viral infection cycles in nonmammalian contexts e.g., mycoviruses, v) imaging patient derived tissue pathologies, and vi) exploring the emergence of eukaryotes through ancient, self-organizing multicellular communities such as slime molds.

We are poised to enter an era of cryo-ET discovery of cellular interactions in real space, however, advances in culturing methods will be necessary. For example, many multispecies communities important to ecological functioning (such as lichens and seaweeds) are not easily cultured in labs or in liquid media compatible with freezing. Concentrated salt water has a depressed freezing point which hinders sample vitrification (77) and methods do not currently exist for growing mineral associated communities on grids. Development of novel culturing methods, paired with lift-out techniques and customized grids, is crucial to accelerate cryo-ET discovery across biodiverse lineages.

Finally, coupling cryo-ET data with additional contextualizing modalities will increase the functional impact of structural cell biology (78, 79). Molecular context can be gained through conjugated gold (74, 80), cathodoluminescent labels (81) or genetically encoded fluorophores to pinpoint individual molecules in situ (correlated light and EM, CLEM) (11, 82). Additionally, microfluidics-based mix-and-spray approaches or optogenetic stimulation allow capture of cellular processes with temporal context (time-resolved freezing) (55). This approach was, for example, used to track pH-induced shedding of the *C. crescentus* S-layer (55). These bonus methods add “color” to cryo-ET data, enabling more robust structure–function relationships to emerge than in traditional, label-free tomograms.

Herein, we outline the most comprehensive synthesis of cellular cryo-ET data to date. We identify critical transition points between freezing and sectioning approaches and the underlying factors which shape methodological choice. We provide an exhaustive comparison of the cellular fractions within lamellae across all domains of life and quantitatively evaluate how close current sectioning methods get to imaging whole cells or organisms. We establish a series of guiding principles for cellular cryo-ET research to reduce times and cost associated with imaging, improve cryo-ET accessibility, and facilitate constructions of structural architectures over multicellular communities. Finally, we identify vast clades of life where structural information is lacking and where cryo-ET research can expedite our understanding of cellular behavior in space.

## Supplementary Material

Appendix 01 (PDF)

## Data Availability

Code data have been deposited in Zenodo 10.5281/zenodo.15831544 (83). Study data are included in the article and/or *SI Appendix*.
