# Supplementary material for: Cryogenic electron tomography by the numbers: Charting underexplored lineages in structural cell biology
Source: Proc Natl Acad Sci U S A. 2026 Feb 18;123(8):e2518350123. doi: 10.1073/pnas.2518350123 (PMC12933124; doi:10.1073/pnas.2518350123)
Supplement: Supplementary file 1 — Appendix 01 (PDF) [file pnas.2518350123.sapp.pdf]

Supplementary Information:

Cryogenic Electron Tomography by the Numbers: Charting Underexplored Lineages in Structural Cell Biology

T. Bertie Ansell[1,2,], Louis Berrios[1], Kabir Peay[1,3], Peter Dahlberg[2]
[1] Department of Biology, Stanford University, Stanford, CA 94305, USA
[2] Division of Cryo-EM and Bioimaging, SLAC National Accelerator Laboratory, Menlo Park, CA 94025, USA
[3] Department of Earth System Science, Stanford University, Stanford, CA 94305, USA

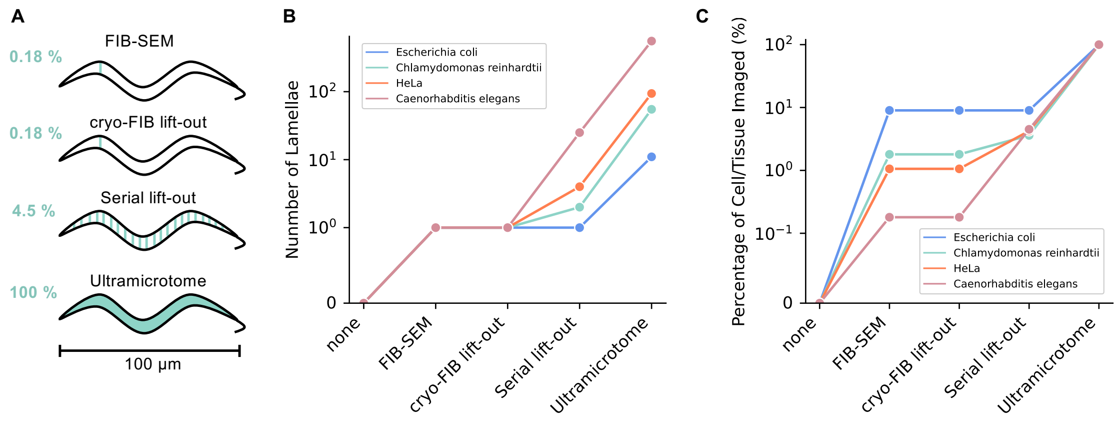

Supplementary Figure 1: The fraction of cellular material captured within lamellae and by different sectioning methods.
A) Schematic of percentage of cellular material captured within lamellae for each sectioning method applied to *C. elegens*. Teal lines indicate individual lamellae. **B)** Number of lamellae which could be produced by following each sectioning approach to completion once, for four model cells/tissues (*E. coli*: blue, *C. reinhardtii*: teal, HeLa: orange, *C. elegens*: red). **C)** Percentage of cell/tissue captured within lamellae by following each sectioning method to completion for the four cells/tissue (defined as in Equation 1).

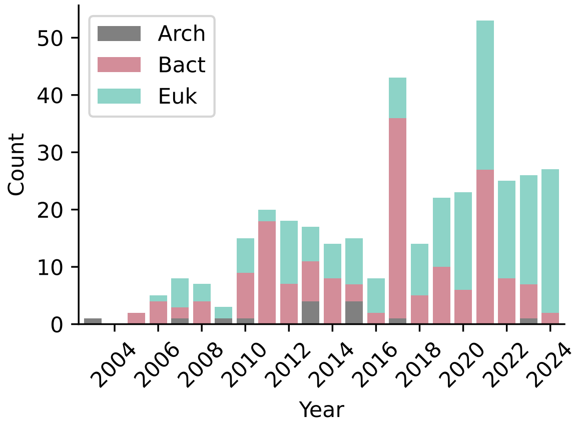

Supplementary Figure 2: Increasing prevalence of eukaryotic cryo-ET.
The number of cellular cryo-ET imaging events by year included within this analysis until the end of 2024 coloured by domain. The proportion of eukaryotic cells imaged increases with time due to recent advances in sample thinning. The limited penetration power of electrons necessitates sectioning cells >300 nm thick. Initially, this was done using the ultramicrotome which can take years of dedication to master. Recent advances in use of FIB-milling (gallium or plasma) have increased throughput, automated milling pipelines and decreased labour constraints, enabling routine imaging of eukaryotic cells by cryo-ET.

Supplementary Table 1: Cells and tissues studied by cryo-ET included within this analysis.

Cell type	Domain	Freezing method	Sectioning method	Cryoprotectant	Cell Width/ Thickness (µm)	Cell Length (µm)	Cell Length-2 (µm)	Dimensions Citation (PMID, DOI, URL)	Percentage of Cell/Tissue Imaged (%)	Shape	Title	DOI
Aplasma	Arch	Plunge	none		0.6	0.7		23865623	25.71	sphere	[1]	10.1186/1471-2164-14-485
ARMAN	Arch	Plunge	none		0.2	0.3		18946497	60.00	sphere	[2]	10.1038/ismej.2008.99
Eplasma	Arch	Plunge	none		0.6	0.7		23865623	25.71	sphere	[1]	10.1186/1471-2164-14-485
Ferroplasma	Arch	Plunge	none		0.6	0.7		23865623	25.71	sphere	[1]	10.1186/1471-2164-14-485
Halobacterium salinarum	Arch	Plunge	none		0.9	1.4		34594449	12.86	capsule	[3]	10.1111/1758-2229.12265
Haloquadratum walsbyi	Arch	Plunge	none		1.2	1.2	0.2	34594449	15.00	rectangle	[4]	10.1099/ijs.0.64690-0
Methanoregula formicica	Arch	Plunge	none		0.4	2		34594449	9.00	capsule	[3]	10.1111/1758-2229.12265
Methanospirillum hungatei	Arch	Plunge	none		0.5	7		39671499	2.57	capsule	[5]	10.1038/s41467-023-42368-2
Methanospirillum hungatei	Arch	Plunge	none		0.4	7	0.4	39671499	2.57	rectangle	[3]	10.1111/1758-2229.12265
Pyrodictium abyssi	Arch	Plunge	none		0.5	1.5		10438790	12.00	cone	[6]	10.1016/S1047-8477(02)00581-6
Sulfolobus acidocaldarius	Arch	Plunge	none		1.3	1.6		34594449	11.25	capsule	[7]	10.1091/mbc.E12-11-0785
Sulfolobus solfataricus	Arch	Plunge	none		1.6	1.6		21134637	11.25	sphere	[8]	10.1016/j.str.2010.10.005
Thermococcus kodakarensis	Arch	Plunge	none		1.2	1.2		34594449	15.00	sphere	[3]	10.1111/1758-2229.12265
Thermococcus kodakarensis	Arch	Plunge	none		1.2	1.2		34594449	15.00	sphere	[9]	10.15252/embr.201744070
Acetonema longum	Bact	Plunge	none		0.3	6		34594449	3.00	capsule	[10]	10.1128/mBio.00298-21
Acetonema longum	Bact	Plunge	none		0.3	6		34594449	3.00	capsule	[11]	10.1038/emboj.2011.186
Acetonema longum	Bact	Plunge	none		0.3	6		34594449	3.00	capsule	[12]	10.1016/j.str.2019.01.005

Acetonema longum (APO-1)	Bact	Plunge	none		0.3	6		34594449	3.00	capsule	[13]	10.1128/JB.00712-13
Acetonema longum (APO-1)	Bact	Plunge	none		0.3	6		34594449	3.00	capsule	[14]	10.1016/j.cell.2011.07.029
Acinetobacter baumannii (RUH 30233T)	Bact	Plunge	Ultramicrotome	20% glycerol	1.25	2		29686523	9.00	capsule	[15]	10.1016/j.jsb.2012.12.004
Agrobacterium tumetaciens	Bact	Plunge	none		0.8	1.5		34594449	12.00	capsule	[16]	10.1128/JB.00371-20
Amoebophilus asiaticus	Bact	Plunge	FIB-SEM		0.55	0.8		34594449	22.50	capsule	[17]	10.1126/science.aan7904
Anabaena sp.	Bact	Plunge	FIB-SEM		4	8		39271453	2.25	capsule	[18]	10.1016/j.cell.2019.05.055
Azospirillum brasilense	Bact	Plunge	none		0.7	2		doi.org/10.1007/s00374-024-01839-4	9.00	capsule	[19]	10.1128/JB.00100-17
Bacillus cereus	Bact	Plunge	none		0.75	3		34594449	6.00	capsule	[13]	10.1128/JB.00712-13
Bacillus subtilis	Bact	Plunge	none		0.75	3		34594449	6.00	capsule	[20]	10.1111/mmi.12201
Bacillus subtilis	Bact	Plunge	none		0.75	3		34594449	6.00	capsule	[13]	10.1128/JB.00712-13
Bacillus subtilis	Bact	Plunge	none		0.75	3		34594449	6.00	capsule	[10]	10.1128/mBio.00298-21
Bacillus subtilis	Bact	Plunge	FIB-SEM		0.75	3		34594449	6.00	capsule	[21]	10.1016/j.cell.2018.01.027
Bacillus subtilis (minicell)	Bact	Plunge	none		0.6	0.6		27480510	30.00	sphere	[22]	10.1016/j.jsb.2016.07.019
Bacillus thuringiensis	Bact	Plunge	none		0.75	3		34594449	6.00	capsule	[13]	10.1128/JB.00712-13
Bdellovibrio bacteriovorus	Bact	Plunge	none		0.4	1.4		34594449	12.86	capsule	[19]	10.1128/JB.00100-17
Bdellovibrio bacteriovorus	Bact	Plunge	none		0.4	1.4		34594449	12.86	capsule	[23]	10.1038/s41564-023-01401-2
Bdellovibrio bacteriovorus	Bact	Plunge	none		0.4	1.4		34594449	12.86	capsule	[24]	10.1016/j.jsb.2018.12.008
Bdellovibrio bacteriovorus	Bact	Plunge	none		0.4	1.4		34594449	12.86	capsule	[12]	10.1016/j.str.2019.01.005
Bdellovibrio bacteriovorus	Bact	Plunge	none		0.4	1.4		34594449	12.86	capsule	[24]	10.1016/j.jsb.2018.12.008
Belliella baltica	Bact	Plunge	none		0.7	2.3		28438890	7.83	capsule	[25]	10.15252/embj.201696235
Borrelia afzelii	Bact	Plunge	none		0.22	20		7043737	0.90	capsule	[26]	10.1016/j.jsb.2009.08.008
Borrelia burgdorferi	Bact	Plunge	none		0.22	20		7043737	0.90	capsule	[27]	10.1111/j.1462-5822.2011.01571.x

Borrelia burgdorferi	Bact	Plunge	none		0.22	20		7043737	0.90	capsule	[28]	10.1016/j.jsb.2023.107990
Borrelia burgdorferi	Bact	Plunge	none		0.22	20		7043737	0.90	capsule	[19]	10.1128/JB.00100-17
Borrelia burgdorferi	Bact	Plunge	none		0.22	20		7043737	0.90	capsule	[29]	10.7554/eLife.73099
Borrelia burgdorferi	Bact	Plunge	none		0.22	20		7043737	0.90	capsule	[29]	10.1038/emboj.2011.186
Borrelia burgdorferi	Bact	Plunge	none		0.22	20		7043737	0.90	capsule	[26]	10.1016/j.jsb.2009.08.008
Borrelia garinii	Bact	Plunge	none		0.22	20		7043737	0.90	capsule	[27]	10.1111/j.1462-5822.2011.01571.x
Borrelia garinii	Bact	Plunge	none		0.22	20		7043737	0.90	capsule	[26]	10.1016/j.jsb.2009.08.008
Brucella abortus	Bact	Plunge	none		0.7	1.4		34594449	12.86	capsule	[19]	10.1128/JB.00100-17
Campylobacter jejuni	Bact	Plunge	none		0.25	1.2		34594449	15.00	capsule	[19]	10.1128/JB.00100-17
Campylobacter jejuni	Bact	Plunge	none		0.25	1.2		34594449	15.00	capsule	[30]	10.1002/mbo3.200
Campylobacter jejuni	Bact	Plunge	none		0.25	1.2		34594449	15.00	capsule	[10]	10.1128/mBio.00298-21
Campylobacter jejuni	Bact	Plunge	none		0.25	1.2		34594449	15.00	capsule	[11]	10.1038/emboj.2011.186
Caulobacter crescentus	Bact	Plunge	none	30% ethylene glycol	0.8	2.75		25778096	6.55	capsule	[31]	10.1073/pnas.2001849117
Caulobacter crescentus	Bact	Plunge	none		0.8	2.75		25778096	6.55	capsule	[32]	10.1016/j.jsb.2011.10.004
Caulobacter crescentus	Bact	Plunge	none		0.8	2.75		25778096	6.55	capsule	[33]	10.1016/j.jsb.2022.107901
Caulobacter crescentus	Bact	Plunge	none		0.8	2.75		25778096	6.55	capsule	[34]	10.1016/j.jsb.2022.107881
Caulobacter crescentus	Bact	Plunge	none		0.8	2.75		25778096	6.55	capsule	[35]	10.1091/mbc.E24-01-0042
Caulobacter crescentus	Bact	Plunge	none		0.8	2.75		25778096	6.55	capsule	[36]	10.1073/pnas.0808035105
Caulobacter crescentus	Bact	Plunge	none		0.8	2.75		25778096	6.55	capsule	[19]	10.1128/JB.00100-17
Caulobacter crescentus	Bact	Plunge	none		0.8	2.75		25778096	6.55	capsule	[29]	10.7554/eLife.73099
Caulobacter crescentus	Bact	Plunge	none		0.8	2.75		25778096	6.55	capsule	[37]	10.1038/sj.emboj.7601895
Caulobacter crescentus	Bact	Plunge	none		0.8	2.75		25778096	6.55	capsule	[11]	10.1038/emboj.2011.186

Caulobacter crescentus	Bact	Plunge	none		0.8	2.75		25778096	6.55	capsule	38	10.1016/j.cell.2019.12.006
Caulobacter crescentus	Bact	Plunge	none		0.8	2.75		25778096	6.55	capsule	39	10.1016/j.jsb.2005.08.004
Caulobacter crescentus (NA1000)	Bact	Plunge	none		0.8	2.75		25778096	6.55	capsule	25	10.15252/embj.201696235
Chitinophaga pinensis	Bact	Plunge	none		0.65	40		21304681	0.45	capsule	29	10.7554/eLife.73099
Chlamydia trachomatis	Bact	Plunge	none		0.4	0.4		24809274	45.00	sphere	40	10.1111/cmi.12310
Deinococcus radiodurans	Bact	Plunge	FIB-SEM		1.5	1.5		34977598	12.00	sphere	41	10.1016/j.crstbi.2021.12.001
Delftia acidovorans	Bact	Plunge	none		0.8	0.8		34594449	22.50	sphere	29	10.7554/eLife.73099
Escherichia coli	Bact	Plunge	FIB-SEM		1	2		2403552, 24287933	9.00	capsule	42	10.1016/j.jsb.2021.107743
Escherichia coli	Bact	HPF	FIB-SEM		1	2		2403552, 24287933	9.00	capsule	43	10.1038/s41467-022-29501-3
Escherichia coli	Bact	Plunge	none		1	2		2403552, 24287933	9.00	capsule	19	10.1128/JB.00100-17
Escherichia coli	Bact	Plunge	none		1	2		2403552, 24287933	9.00	capsule	11	10.1038/emboj.2011.186
Escherichia coli	Bact	Plunge	none		1	2		2403552, 24287933	9.00	capsule	44	10.1038/emboj.2012.76
Escherichia coli	Bact	Plunge	none		1	2		2403552, 24287933	9.00	capsule	45	10.1016/j.str.2010.12.002
Escherichia coli	Bact	Plunge	none		1	2		2403552, 24287933	9.00	capsule	46	10.1016/j.jsb.2017.11.002
Escherichia coli	Bact	Plunge	none	10% Ficoll PM 70	1	2		2403552, 24287933	9.00	capsule	47	10.1371/journal.pbio.1001213
Escherichia coli	Bact	Plunge	none		1	2		2403552, 24287933	9.00	capsule	10	10.1128/mBio.00298-21
Escherichia coli	Bact	Plunge	FIB-SEM		1	2		2403552, 24287933	9.00	capsule	48	10.1016/j.jsb.2024.108097
Escherichia coli	Bact	Plunge	FIB-SEM		1	2		2403552, 24287933	9.00	capsule	49	10.1016/j.jsb.2022.107911
Escherichia coli	Bact	Plunge	FIB-SEM		1	2		2403552, 24287933	9.00	capsule	50	10.1016/j.jsb.2016.02.013
Escherichia coli (1094)	Bact	Plunge	FIB-SEM		1	2		2403552, 24287933	9.00	capsule	16	10.1128/JB.00371-20
Escherichia coli (BL21)	Bact	Plunge	FIB-SEM		1	2		2403552, 24287933	9.00	capsule	51	10.15252/embj.2018100886
Escherichia coli (K12)	Bact	Plunge	FIB-SEM		1	2		2403552, 24287933	9.00	capsule	52	10.1016/j.jsb.2012.07.003

Escherichia coli (K12)	Bact	Plunge	none		1	2		2403552, 24287933	9.00	capsule	53	10.1016/j.str.2014.09.017
Escherichia coli (MG1655, XL-10)	Bact	Plunge	none		1	2		2403552, 24287933	9.00	capsule	36	10.1073/pnas.0808035105
Escherichia coli (MG1655)	Bact	Plunge	FIB-SEM		1	2		2403552, 24287933	9.00	capsule	54	10.1038/s41564-022-01210-z
Escherichia coli (mini cells)	Bact	Plunge	none		0.25	0.25		2838458	72.00	sphere	55	10.1021/acsami.9b01826
Escherichia coli (mini cells)	Bact	Plunge	none		0.25	0.25		2838458	72.00	sphere	56	10.1016/j.cell.2018.10.033
Escherichia coli (RP437)	Bact	Plunge	none		1	2		2403552, 24287933	9.00	capsule	57	10.1038/emboj.2011.77
Flavobacterium anhuiense	Bact	Plunge	none		0.3	1.75		18398165	10.29	capsule	29	10.7554/eLife.73099
Flavobacterium johnsoniae	Bact	Plunge	none		0.5	3		33441737	6.00	capsule	29	10.7554/eLife.73099
Geobacter sulfurreducens	Bact	Plunge	none		0.5	2		10.1080/01490451.2015.1099765	9.00	capsule	58	10.1038/s41522-023-00384-6
Gluconacetobacter hansenii	Bact	Plunge	FIB-SEM		0.6	2		10.1007/s13213-011-0288-4	9.00	capsule	16	10.1128/JB.00371-20
Gluconacetobacter xylinus	Bact	Plunge	none		0.6	2		10.1007/s13213-011-0288-4	9.00	capsule	16	10.1128/JB.00371-20
Halothiobacillus neapolitanus	Bact	Plunge	none		0.45	1.8		34594449	10.00	capsule	59	10.1016/j.jmb.2009.11.019
Halothiobacillus neapolitanus	Bact	Plunge	none		0.45	1.8		34594449	10.00	capsule	19	10.1128/JB.00100-17
Halothiobacillus neapolitanus (c2)	Bact	Plunge	none		0.45	1.8		34594449	10.00	capsule	25	10.15252/embj.201696235
Helicobacter hepaticus	Bact	Plunge	none		0.3	5		34594449	3.60	capsule	29	10.7554/eLife.73099
Helicobacter hepaticus	Bact	Plunge	none		0.3	5		34594449	3.60	capsule	10	10.1128/mBio.00298-21
Helicobacter hepaticus	Bact	Plunge	none		0.3	5		34594449	3.60	capsule	11	10.1038/emboj.2011.186
Helicobacter pylori	Bact	Plunge	none		0.5	3.5		8903168	5.14	capsule	60	10.1073/pnas.2118401119
Helicobacter pylori	Bact	Plunge	none		0.5	3.5		8903168	5.14	capsule	61	10.1016/j.celrep.2018.03.085
Helicobacter pylori	Bact	Plunge	none		0.5	3.5		8903168	5.14	capsule	19	10.1128/JB.00100-17
Helicobacter pylori	Bact	Plunge	none		0.5	3.5		8903168	5.14	capsule	29	10.7554/eLife.73099
Hydrogenovibrio crunogenus	Bact	Plunge	none		0.5	1.5		34594449	12.00	capsule	10	10.1128/mBio.00298-21
Hylemonella gracilis	Bact	Plunge	none		0.2	6		34594449	3.00	capsule	19	10.1128/JB.00100-17

Hylemonella gracilis	Bact	Plunge	none		0.2	6		34594449	3.00	capsule	29	10.7554/eLife.73099
Hylemonella gracilis	Bact	Plunge	none		0.2	6		34594449	3.00	capsule	10	10.1128/mBio.00298-21
Hylemonella gracilis	Bact	Plunge	none		0.2	6		34594449	3.00	capsule	11	10.1038/emboj.2011.186
Hylemonella gracilis	Bact	Plunge	none		0.2	6		34594449	3.00	capsule	12	10.1016/j.str.2019.01.005
Hyphomonas neptunium	Bact	Plunge	none		1	1.8		34594449	10.00	capsule	19	10.1128/JB.00100-17
Hyphomonas neptunium	Bact	Plunge	none		1	1.8		34594449	10.00	capsule	11	10.1038/emboj.2011.186
Legionella pneumophila	Bact	Plunge	none		0.5	2		34594449	9.00	capsule	62	10.15252/embr.201643598
Legionella pneumophila	Bact	Plunge	none		0.5	2		34594449	9.00	capsule	63	10.1038/s41564-019-0427-4
Legionella pneumophila (Lp02)	Bact	Plunge	none		0.5	2		34594449	9.00	capsule	25	10.15252/embj.201696235
Lysinibacillus sphaericus	Bact	Plunge	none		0.6	1.5		22248454	12.00	capsule	64	10.1016/j.jsb.2012.01.003
Magnetospirillum gryphiswaldense	Bact	Plunge	none		0.6	2.6		34594449	6.92	capsule	65	10.1016/j.jsb.2015.03.004
Magnetospirillum magnetotacticum	Bact	Plunge	none		0.6	2.6		34594449	6.92	capsule	29	10.7554/eLife.73099
Magnetospirillum magnetotacticum (AMB-1)	Bact	Plunge	none		0.6	2.6		34594449	6.92	capsule	66	10.1128/mBio.01898-15
Magnetovibrio blakemorei (MV-1)	Bact	Plunge	none		0.3	1.5		23246783	12.00	capsule	67	10.1016/j.jsb.2012.12.002
Mesoplasma florum	Bact	Plunge	none		0.5	0.5		38404664	36.00	sphere	68	10.1016/j.jsb.2005.12.004
Mesoplasma florum	Bact	Plunge	none		0.5	0.5		38404664	36.00	sphere	69	10.1016/j.jsb.2005.07.004
Methylomicrobium alcaliphilum	Bact	Plunge	none		1	1.2		34594449	15.00	capsule	70	10.1038/s41467-020-15736-5
Methyloprofundus sedimenti	Bact	Plunge	none		1.2	1.7		34594449	10.59	capsule	71	10.1111/mmi.13553
Mycobacterium smegmatis	Bact	Plunge	none		0.5	2.5		34594449	7.20	capsule	19	10.1128/JB.00100-17
Mycobacterium smegmatis	Bact	Plunge	Ultramicrotome		0.5	2.5		34594449	7.20	capsule	72	10.1016/j.jsb.2010.02.011
Mycoplasma genitalium	Bact	Plunge	none		0.3	1		34594449	18.00	capsule	73	10.1016/j.jsb.2014.05.007
Mycoplasma pneumoniae	Bact	Plunge	none		0.3	1		34594449	18.00	capsule	74	10.1111/j.1365-2958.2006.05113.x

Mycoplasma pneumoniae	Bact	Plunge	none		0.3	1		34594449	18.00	capsule	75	10.1016/j.jsb.2006.04.010
Myxococcus xanthus	Bact	Plunge	none	5% Ficoll PM 70, 10% Ficoll PM 70 and 10% ethylene glycol	0.7	5		34594449	3.60	capsule	76	10.1038/NMETH.2961
Myxococcus xanthus	Bact	Plunge	none		0.7	5		34594449	3.60	capsule	19	10.1128/JB.00100-17
Myxococcus xanthus	Bact	Plunge	none		0.7	5		34594449	3.60	capsule	29	10.7554/eLife.73099
Myxococcus xanthus	Bact	Plunge	none		0.7	5		34594449	3.60	capsule	77	10.15252/embr.201744072
Parachlamydia acanthamoebae	Bact	Plunge	none		0.5	0.5		8979345	36.00	sphere	78	10.1111/1462-2920.12299
Prochlorococcus marinus	Bact	Plunge	none		0.5	0.7		12917486	25.71	capsule	79	10.1038/nsmb.1823
Prosthecobacter debontii	Bact	Plunge	none		0.4	5		9296261	3.60	capsule	19	10.1128/JB.00100-17
Prosthecobacter fluviatilus	Bact	Plunge	none		0.5	5		18599695	3.60	capsule	19	10.1128/JB.00100-17
Prosthecobacter vanneervenii	Bact	Plunge	none	10% Ficoll PM 70	0.4	8		9296261	2.25	capsule	47	10.1371/journal.pbio.1001213
Prosthecobacter vanneervenii	Bact	Plunge	none		0.4	8		9296261	2.25	capsule	19	10.1128/JB.00100-17
Proteus mirabilis	Bact	Plunge	none		1	2		15213138	9.00	capsule	80	10.3390/ijms24098292
Proteus mirabilis	Bact	Plunge	none		1	2		15213138	9.00	capsule	25	10.15252/embj.201696235
Protochlamydia amoebophila	Bact	Plunge	none		0.8	0.8		24292151	22.50	sphere	78	10.1111/1462-2920.12299
Pseudoalteromonas luteoviolacea	Bact	Plunge	none		0.6	1.5		34594449	12.00	capsule	29	10.7554/eLife.73099
Pseudomonas aeruginosa	Bact	Plunge	none		0.8	2		34594449	9.00	capsule	70	10.1038/s41467-020-15736-5
Pseudomonas deceptionensis	Bact	Plunge	none		0.8	2		34594449	9.00	capsule	81	10.1016/j.jsb.2015.01.008
Pseudomonas deceptionensis	Bact	HPF	Ultramicrotome	30% dextran	0.8	2		34594449	9.00	capsule	81	10.1016/j.jsb.2015.01.008
Ralstonia eutropha	Bact	Plunge	none		0.7	1.2		22178974	15.00	capsule	25	10.15252/embj.201696235
Ralstonia eutropha	Bact	Plunge	none		0.7	1.2		22178974	15.00	capsule	19	10.1128/JB.00100-17
Ralstonia eutropha (H16)	Bact	Plunge	none		0.7	1.2		22178974	15.00	capsule	82	10.1128/JB.06125-11
Rectangular bacterial cells	Bact	Plunge	none		5.08	3.95		37055390	4.56	rectangle	83	10.1016/j.xpro.2022.101658
Rhodopseudomonas viridis	Bact	Plunge	none		0.3	1.6		36296300	11.25	capsule	84	10.1016/j.jsb.2007.09.014

Salmonella enterica	Bact	Plunge	none		0.5	0.5		24284544	36.00	sphere	11	10.1038/emboj.2011.186
Salmonella enterica (mini cells)	Bact	Plunge	none		0.5	0.5		24284544	36.00	sphere	28	10.1016/j.jsb.2023.107990
Salmonella enterica (mini cells)	Bact	Plunge	none		0.5	0.5		24284544	36.00	sphere	85	10.1016/j.cell.2017.02.022
Salmonella enterica (minicell)	Bact	Plunge	none		0.5	0.5		24284544	36.00	sphere	25	10.15252/embj.201696235
Shewanella oneidensis	Bact	Plunge	none		0.6	2		34594449	9.00	capsule	86	10.1073/pnas.1718810115
Shewanella oneidensis	Bact	Plunge	none		0.6	2		34594449	9.00	capsule	70	10.1038/s41467-020-15736-5
Shewanella oneidensis	Bact	Plunge	none		0.6	2		34594449	9.00	capsule	29	10.7554/eLife.73099
Shewanella putrifaciens	Bact	Plunge	none		0.6	2		34594449	9.00	capsule	19	10.1128/JB.00100-17
Simkania negevensis	Bact	Plunge	none		0.7	0.7		34594449	25.71	sphere	78	10.1111/1462-2920.12299
Synechococcus sp.	Bact	Plunge	FIB-SEM		0.8	1.5		25662459	12.00	capsule	87	10.1016/j.cell.2021.05.011
Tetrasphaera remsis	Bact	Plunge	none		0.6	0.8		34594449	22.50	capsule	88	10.1099/ijs.0.65137-0
Thiomicrospira crunogena	Bact	Plunge	none		0.4	1.5		10.1099/00207713-35-4-422	12.00	capsule	59	10.1016/j.jmb.2009.11.019
Thiomicrospira crunogena	Bact	Plunge	none		0.4	1.5		10.1099/00207713-35-4-422	12.00	capsule	19	10.1128/JB.00100-17
Thiomonas intermedia	Bact	Plunge	none		0.55	1.4		34594449	12.86	capsule	59	10.1016/j.jmb.2009.11.019
Thiomonas intermedia	Bact	Plunge	none		0.55	1.4		34594449	12.86	capsule	19	10.1128/JB.00100-17
Thiomonas intermedia	Bact	Plunge	none		0.55	1.4		34594449	12.86	capsule	25	10.15252/embj.201696235
Treponema denticola	Bact	Plunge	none		0.25	5.5		34594449	3.27	capsule	89	10.1016/j.jsb.2008.03.009
Treponema denticola	Bact	Plunge	none		0.25	5.5		34594449	3.27	capsule	90	10.1111/cmi.12886
Treponema primitia	Bact	Plunge	none		0.25	5.5		34594449	3.27	capsule	91	10.1038/nature05015
Treponema primitia	Bact	Plunge	none		0.25	5.5		34594449	3.27	capsule	11	10.1038/emboj.2011.186
Vibrio cholerae	Bact	Plunge	none		0.8	2.5		26933214	7.20	capsule	92	10.1038/s42003-021-02962-w
Vibrio cholerae	Bact	Plunge	none		0.8	2.5		26933214	7.20	capsule	93	10.1038/nature10846
Vibrio cholerae	Bact	Plunge	none		0.8	2.5		26933214	7.20	capsule	19	10.1128/JB.00100-17

Vibrio cholerae	Bact	Plunge	none		0.8	2.5		26933214	7.20	capsule	70	10.1038/s41467-020-15736-5
Vibrio cholerae	Bact	Plunge	none		0.8	2.5		26933214	7.20	capsule	11	10.1038/emboj.2011.186
Yersinia enterocolitica	Bact	Plunge	none		0.65	2		22019131	9.00	capsule	32	10.1016/j.jsb.2011.10.004
Yersinia enterocolitica	Bact	Plunge	FIB-SEM		0.65	2		22019131	9.00	capsule	94	10.1016/j.jsb.2021.107701
SK-MEL-2 (human melanoma)	Euk	Plunge	none		7	25		36133718	0.72	cone	95	10.1016/j.devcel.2022.04.012
17Cl-1 (mouse)	Euk	Plunge	FIB-SEM		7	35		31536774	0.51	cone	96	10.1016/j.jsb.2019.09.006
Anncaliia algerae	Euk	HPF	FIB-SEM		2	3		31332877	6.00	capsule	43	10.1038/s41467-022-29501-3
Anncaliia algerae (microsporidia tubule region)	Euk	Plunge	none		0.1	100		31332877	0.18	capsule	97	10.1111/jeu.12751
BSC-1 (monkey kidney)	Euk	Plunge	FIB-SEM		7	30		11222860	0.60	cone	98	10.1038/s41596-020-0320-x
BSC-1 (monkey kidney)	Euk	Plunge	none		7	30		11222860	0.60	cone	99	10.1016/j.jsb.2024.108163
Caenorhabditis elegans	Euk	HPF	FIB-SEM	2-methyl pentane	50	1000		25961413	0.02	capsule	100	10.1038/NMETH.3401
Caenorhabditis elegans	Euk	HPF	Serial lift-out, FIB-SEM	20% Ficoll 400	50	1000		25961413	0.02	capsule	101	10.1038/s41592-023-02113-5
Caenorhabditis elegans	Euk	HPF	Cryo-FIB lift-out, FIB-SEM	10% dextran	50	1000		25961413	0.02	capsule	102	10.1016/j.jsb.2015.07.012
Caenorhabditis elegans	Euk	HPF	FIB-SEM	2-methylpentane	50	1000		25961413	0.02	capsule	103	10.1111/boc.202400064
Caenorhabditis elegans (embrios)	Euk	Plunge	FIB-SEM		30	50		25961413	0.36	sphere	100	10.1038/NMETH.3401
Chlamydomonas reinhardtii	Euk	Plunge	FIB-SEM		8	10		11337403	1.80	capsule	104	10.1038/s41592-021-01275-4
Chlamydomonas reinhardtii	Euk	Plunge	FIB-SEM		8	10		11337403	1.80	capsule	105	10.7554/eLife.7050610.7554/eLife.70506.sa010.7554/eLife.70506.sa110.7554/eLife.70506.sa2
Chlamydomonas reinhardtii	Euk	Plunge	none		8	10		11337403	1.80	capsule	106	10.1083/jcb.200903082
Chlamydomonas reinhardtii	Euk	Plunge	FIB-SEM		8	10		11337403	1.80	capsule	107	10.1016/j.jsb.2016.07.010

Chlamydomonas reinhardtii	Euk	Plunge	none		8	10		11337403	1.80	capsule	[108]	10.1038/s41556-018-0213-1
Chlamydomonas reinhardtii	Euk	Plunge	none		8	10		11337403	1.80	capsule	[109]	10.1038/ncb2939
Chlamydomonas reinhardtii	Euk	Plunge	FIB-SEM		8	10		11337403	1.80	capsule	[87]	10.1016/j.cell.2021.05.011
Chlamydomonas reinhardtii	Euk	Plunge	none		8	10		11337403	1.80	capsule	[110]	10.1038/s41594-022-00905-5
Chlamydomonas reinhardtii	Euk	Plunge	none		8	10		11337403	1.80	capsule	[111]	10.15252/embj.2020106246
Chlamydomonas reinhardtii	Euk	Plunge	FIB-SEM		8	10		11337403	1.80	capsule	[112]	10.1091/mbc.E19-03-0141
Chlamydomonas reinhardtii	Euk	Plunge	FIB-SEM		8	10		11337403	1.80	capsule	[113]	10.1016/j.jsb.2023.108005
CHO (chinese hamsper ovary)	Euk	HPF	Ultramicrotome		7	15		17189170	1.20	cone	[114]	10.1042/BC20060081
CHO (chinese hamsper ovary)	Euk	Plunge	none		7	15		17189170	1.20	cone	[115]	10.1242/jcs.073486
Dictyostelium discoideum	Euk	Plunge	Ultramicrotome		7	12		8937986	1.50	cone	[72]	10.1016/j.jsb.2010.02.011
Dictyostelium discoideum	Euk	Plunge	none		7	12		8937986	1.50	cone	[116]	10.1016/j.jsb.2011.08.012
Dictyostelium discoideum	Euk	Plunge	FIB-SEM		7	12		8937986	1.50	cone	[117]	10.1016/j.str.2016.05.004
Dictyostelium discoideum	Euk	Plunge	FIB-SEM		7	12		8937986	1.50	cone	[118]	10.1016/j.str.2019.05.009
Drosophila melanogaster (eggs)	Euk	HPF	Cryo-FIB lift-out, FIB-SEM	20% Ficoll 70	180	510		19032497	0.04	sphere	[105]	10.7554/eLife.7050610.7554/eLife.70506.sa010.7554/eLife.70506.sa110.7554/eLife.70506.sa2
Drosophila melanogaster (embrios)	Euk	HPF	FIB-SEM	2-methyl pentane	180	510		19032497	0.04	sphere	[100]	10.1038/NMETH.3401
Drosophila melanogaster (Kenyon brain cells)	Euk	HPF	Ultramicrotome	20% dextran	100	1000	200	25301679	0.02	sphere	[119]	10.1016/j.jsb.2014.09.012
Drosophila melanogaster (S2 cells)	Euk	Plunge	none	0.125% DMSO	7	11.7		29907103	1.54	cone	[120]	10.15252/embr.202357264
Encephalitozoon hellem	Euk	HPF	FIB-SEM		2	4		35387991	4.50	sphere	[43]	10.1038/s41467-022-29501-3
Gephyrocapsa huxleyi	Euk	Plunge	FIB-SEM		3	3		31530807	6.00	sphere	[105]	10.7554/eLife.7050610.7554/eLife.70506.sa010.7554/eLif

												e.70506.sa110.7554/eLife.70506.sa2
HAP1 (human leukemia)	Euk	Plunge	none		7	11		33184093	1.64	cone	121	10.1083/jcb.201911154
HEK293	Euk	HPF	Ultramicrotome		7	14		https://bionumbers.hms.harvard.edu/files/Sizes%20of%20various%20cells.pdf	1.29	cone	122	10.1038/srep13017
HEK293	Euk	Plunge	FIB-SEM		7	14		https://bionumbers.hms.harvard.edu/files/Sizes%20of%20various%20cells.pdf	1.29	cone	98	10.1038/s41596-020-0320-x
HEK293	Euk	Plunge	FIB-SEM	10% glycerol	7	14		https://bionumbers.hms.harvard.edu/files/Sizes%20of%20various%20cells.pdf	1.29	cone	123	10.1016/j.molcel.2024.04.018
HEK293 (HEK293S)	Euk	HPF	FIB-SEM		7	14		https://bionumbers.hms.harvard.edu/files/Sizes%20of%20various%20cells.pdf	1.29	cone	43	10.1038/s41467-022-29501-3
HEK293 (HEK293T)	Euk	Plunge	none		7	14		https://bionumbers.hms.harvard.edu/files/Sizes%20of%20various%20cells.pdf	1.29	cone	124	10.1128/jvi.00368-24
HEK293 (HEK293T)	Euk	Plunge	none		7	14		https://bionumbers.hms.harvard.edu/files/Sizes%20of%20various%20cells.pdf	1.29	cone	125	10.1016/j.cell.2021.01.033
HEK293 (HEK293T)	Euk	Plunge	FIB-SEM		7	14		https://bionumbers.hms.harvard.edu/files/Sizes%20of%20various%20cells.pdf	1.29	cone	126	10.1016/j.cell.2020.08.004
HEK293 (HEK293T)	Euk	Plunge	FIB-SEM		7	14		https://bionumbers.hms.harvard.edu/files/Sizes%20of%20various%20cells.pdf	1.29	cone	127	10.1038/s44318-024-00027-2
HEK293 (HEK293T)	Euk	Plunge	none		7	14		https://bionumbers.hms.harvard.edu/files/Sizes%20of%20various%20cells.pdf	1.29	cone	128	10.1016/j.cell.2024.08.044
HeLa (CCL-2) (human cervical cancer)	Euk	Plunge	Plasma FIB-SEM	glycerol	7	17		5761872	1.06	cone	129	10.1038/s41467-023-36372-9
HeLa (human cervical cancer)	Euk	Plunge	FIB-SEM		7	17		5761872	1.06	cone	52	10.1016/j.jsb.2012.07.003
HeLa (human cervical cancer)	Euk	Plunge	FIB-SEM		7	17		5761872	1.06	cone	105	10.7554/eLife.7050610.7554/eLife.70506.sa010.7554/eLife.70506.sa

												110.7554/eLife.70506.sa2
HeLa (human cervical cancer)	Euk	Plunge	none	EAFS (ethylene glycol, acetamide, Ficoll, sucrose), DES (DMSO, ethylene glycol, sucrose, FCS), DE (DMSO, ethylene glycol, FCS), dextran	7	17		5761872	1.06	cone	130	10.1016/j.bpj.2015.09.029
HeLa (human cervical cancer)	Euk	Plunge	none		7	17		5761872	1.06	cone	131	10.1016/j.jsb.2017.10.009
HeLa (human cervical cancer)	Euk	Plunge	none		7	17		5761872	1.06	cone	132	10.1016/j.jsb.2021.107709
HeLa (human cervical cancer)	Euk	Plunge	none		7	17		5761872	1.06	cone	133	10.1073/pnas.1920323117
HeLa (human cervical cancer)	Euk	Plunge	FIB-SEM		7	17		5761872	1.06	cone	134	10.1038/s41592-022-01749-z
HeLa (human cervical cancer)	Euk	Plunge	FIB-SEM		7	17		5761872	1.06	cone	135	10.1038/s41592-022-01748-0
HeLa (human cervical cancer)	Euk	Plunge	none		7	17		5761872	1.06	cone	136	10.1016/j.str.2011.09.006
HeLa (human cervical cancer)	Euk	Plunge	FIB-SEM		7	17		5761872	1.06	cone	137	10.1016/j.cell.2023.03.015
HeLa (human cervical cancer)	Euk	Plunge	FIB-SEM	DMEM, 10% glycerol	7	17		5761872	1.06	cone	138	10.1016/j.cell.2017.08.009
HeLa (human cervical cancer)	Euk	Plunge	FIB-SEM		7	17		5761872	1.06	cone	107	10.1016/j.jsb.2016.07.010
HeLa (human cervical cancer)	Euk	Plunge	FIB-SEM		7	17		5761872	1.06	cone	139	10.1016/j.jsb.2016.06.020
HeLa (human cervical cancer)	Euk	Plunge	FIB-SEM		7	17		5761872	1.06	cone	140	10.1091/mbc.E18-05-0331
HeLa (human cervical cancer)	Euk	Plunge	none		7	17		5761872	1.06	cone	40	10.1111/cmi.12310
HeLa (human cervical cancer)	Euk	Plunge	none		7	17		5761872	1.06	cone	141	10.1016/j.jsb.2022.107860
HeLa (human cervical cancer)	Euk	Plunge	FIB-SEM		7	17		5761872	1.06	cone	142	10.1016/j.str.2020.05.013
HeLa (human cervical cancer)	Euk	Plunge	FIB-SEM		7	17		5761872	1.06	cone	143	10.1016/j.jsb.2020.107633
HeLa (human cervical cancer)	Euk	Plunge	none		7	17		5761872	1.06	cone	144	10.1016/j.devcel.2024.04.008
HeLa (human cervical cancer)	Euk	Plunge	FIB-SEM		7	17		5761872	1.06	cone	145	10.1038/s41594-024-01218-5
HepG2 (human liver cancer)	Euk	Plunge	FIB-SEM		7	15.5		34884942	1.16	cone	134	10.1038/s41592-022-01749-z

HepG2 (human liver cancer)	Euk	HPF	Ultramicrotome	20% dextran	7	15.5		34884942	1.16	cone	146	10.1016/j.jsb.2013.06.014
HL-1 (mouse cardiomyocyte)	Euk	HPF	Ultramicrotome	20% dextran	7	100	20	9501201	0.18	rectangular based pyramid	147	10.1016/j.jsb.2007.10.008
HT-1080 (human epithelial sarcoma)	Euk	Plunge	none		7	23		24349113	0.78	cone	148	10.1369/0022155415593323
Human brain tissue	Euk	Plunge	FIB-SEM	20% glycerol, 1M trehalose in DPBS	100	2000	2000	38531877	0.01	rectangle	149	10.1038/s41467-024-47066-1
Human primary adiposite cells	Euk	Plunge	none		7	20		29641234	0.90	cone	131	10.1016/j.jsb.2017.10.009
HUVEC (human umbilical vein endothelial cells)	Euk	Plunge	none		7	17		https://www.thermofisher.com/content/dam/LifeTech/migration/en/filelibrary/cell-tissue-analysis/pdfs.par.12078.file.dat/huvec.pdf	1.06	cone	150	10.1038/s41598-019-55766-8
HUVEC (human umbilical vein endothelial cells)	Euk	Plunge	none		7	17		https://www.thermofisher.com/content/dam/LifeTech/migration/en/filelibrary/cell-tissue-analysis/pdfs.par.12078.file.dat/huvec.pdf	1.06	cone	151	10.1016/j.ejcb.2009.07.002
HUVEC (human umbilical vein endothelial cells)	Euk	Plunge	none		7	17		https://www.thermofisher.com/content/dam/LifeTech/migration/en/filelibrary/cell-tissue-analysis/pdfs.par.12078.file.dat/huvec.pdf	1.06	cone	152	10.1016/j.jsb.2021.107791
INS-1E (rat insulinoma beta cells)	Euk	Plunge	none		7	45		29078993	0.40	cone	131	10.1016/j.jsb.2017.10.009
iPSC neurons	Euk	Plunge	none		7	15		20807017	1.20	cone	153	10.1038/s41467-023-36096-w
iPSC neurons	Euk	Plunge	none		7	15		20807017	1.20	cone	154	10.1038/s41556-024-01356-4
Keratinocytes (human)	Euk	Plunge	none		14	14	14	2410922	1.29	rectangle	155	10.1016/j.jsb.2007.07.011
MDA-MB-23 (human epithelial)	Euk	Plunge	none		7	14		10.1088/1361-6439/aa5bbb	1.29	cone	156	10.1016/j.yexcr.2017.05.024
MDCK (canine kidney)	Euk	Plunge	FIB-SEM		7	30		25606673	0.60	cone	98	10.1038/s41596-020-0320-x
MEF (MEF-IRE1a-mNG) (mouse embryonic fibroblasts)	Euk	Plunge	none		3	30	10	34088673	0.60	rectangular based pyramid	157	10.1126/science.abh2474
MEF (mouse embryonic fibroblasts)	Euk	Plunge	Ultramicrotome		3	30	10	34088673	0.60	rectangular based pyramid	15	10.1016/j.jsb.2012.12.004

MEF (mouse embryonic fibroblasts)	Euk	Plunge	FIB-SEM		3	30	10	34088673	0.60	rectangular based pyramid	158	10.1016/j.bpj.2023.07.030
MEF (mouse embryonic fibroblasts)	Euk	Plunge	none		3	30	10	34088673	0.60	rectangular based pyramid	159	10.1242/jcs.120295
MEF (mouse embryonic fibroblasts)	Euk	Plunge	FIB-SEM		3	30	10	34088673	0.60	rectangular based pyramid	160	10.1083/jcb.202204093
MEF (mouse embryonic fibroblasts)	Euk	Plunge	none		3	30	10	34088673	0.60	rectangular based pyramid	161	10.1016/j.str.2020.12.014
MEF (mouse embryonic fibroblasts)	Euk	Plunge	FIB-SEM		3	30	10	34088673	0.60	rectangular based pyramid	50	10.1016/j.jsb.2016.02.013
MEF (mouse embryonic fibroblasts)	Euk	Plunge	FIB-SEM		3	30	10	34088673	0.60	rectangular based pyramid	162	10.1038/s41594-024-01261-2
MEF (mouse embryonic fibroblasts)	Euk	Plunge	FIB-SEM		3	30	10	34088673	0.60	rectangular based pyramid	127	10.1038/s44318-024-00027-2
MEF (mouse embryonic fibroblasts)	Euk	Plunge	FIB-SEM		3	30	10	34088673	0.60	rectangular based pyramid	163	10.1242/jcs.256156
Mouse brain tissue	Euk	HPF	Ultramicrotome	20% w/v 40000 Dextran in NMDG-HEPES solution	60	100	150	37198197	0.18	rectangle	164	10.1039/d2fd00081d
Mouse brain tissue (2mm)	Euk	HPF	Ultramicrotome	20% w/v 40000 Dextran in NMDG-HEPES solution	60	100	150	37198197	0.18	rectangle	165	10.1038/s41467-023-38495-5
Mouse cardiac tissue	Euk	HPF	Ultramicrotome	1-hexadecene, dextran	20	100	30	34174447	0.18	rectangle	166	10.1016/j.jsb.2021.107763
Mouse Islets of Langerhans	Euk	HPF	Cryo-FIB lift-out, FIB-SEM	2-methylpentane	10	100	100	37201639	0.18	rectangle	167	10.1016/j.jsb.2023.107971
Mouse kidney tissue	Euk	HPF	Ultramicrotome	1-hexadecene, dextran	20	100	30	34174447	0.18	rectangle	166	10.1016/j.jsb.2021.107763
Mouse skeletal tissue	Euk	HPF	Ultramicrotome	1-hexadecene, dextran	20	100	30	34174447	0.18	rectangle	166	10.1016/j.jsb.2021.107763
Neurons (Drosophila)	Euk	Plunge	none		4	22	100	https://bionumbers.hms.harvard.edu	0.82	cone	168	10.1083/jcb.202103154
Neurons (mouse hippocampus)	Euk	Plunge	none		4	22	100	https://bionumbers.hms.harvard.edu	0.82	cone	169	10.1242/jcs.259234
Neurons (mouse hippocampus)	Euk	Plunge	none		4	22	100	https://bionumbers.hms.harvard.edu	0.82	cone	170	10.3390/cells11162533
Neurons (mouse primary DRG)	Euk	Plunge	none		4	22	100	https://bionumbers.hms.harvard.edu	0.82	cone	168	10.1083/jcb.202103154
Neurons (mouse primary)	Euk	Plunge	FIB-SEM	DMEM, 10% glycerol	4	22	100	https://bionumbers.hms.harvard.edu	0.82	cone	138	10.1016/j.cell.2017.08.009
Neurons (mouse primary)	Euk	Plunge	none		4	22	100	https://bionumbers.hms.harvard.edu	0.82	cone	171	10.1083/jcb.202106086
Neurons (rat cortical)	Euk	Plunge	FIB-SEM		4	22	100	https://bionumbers.hms.harvard.edu	0.82	cone	172	10.1016/j.cell.2017.12.030
Neurons (rat hippocampus)	Euk	Plunge	none		4	22	100	https://bionumbers.hms.harvard.edu	0.82	cone	65	10.1016/j.jsb.2015.03.004

Neurons (rat hippocampus)	Euk	Plunge	none		4	22	100	https://bionumbers.hms.harvard.edu	0.82	cone	173	10.1016/j.celrep.2019.06.006
Neurons (rat hippocampus)	Euk	Plunge	none		4	22	100	https://bionumbers.hms.harvard.edu	0.82	cone	46	10.1016/j.jsb.2017.11.002
Neurons (rat hippocampus)	Euk	Plunge	none		4	22	100	https://bionumbers.hms.harvard.edu	0.82	cone	174	10.1038/s41421-019-0134-6
Neurons (rat hippocampus)	Euk	Plunge	none		4	22	100	https://bionumbers.hms.harvard.edu	0.82	cone	175	10.1038/s41556-024-01544-2
Neurons (rat primary)	Euk	Plunge	none		4	22	100	https://bionumbers.hms.harvard.edu	0.82	cone	176	10.1016/j.jsb.2007.08.014
Neurons (rat primary)	Euk	Plunge	FIB-SEM		4	22	100	https://bionumbers.hms.harvard.edu	0.82	cone	177	10.15252/embr.202153890
NG108-15 (mouse neuroblastoma/glioma)	Euk	Plunge	none		7	20		12852832	0.90	cone	155	10.1016/j.jsb.2007.07.011
NG108-15 (mouse neuroblastoma/glioma)	Euk	Plunge	none		7	20		12852832	0.90	cone	178	10.1002/cm.21309
NIH/3T3 (mouse embryonic fibroblast)	Euk	Plunge	FIB-SEM		7	15		https://bionumbers.hms.harvard.edu/files/Sizes%20of%20various%20cells.pdf	1.20	cone	98	10.1038/s41596-020-0320-x
NIH/3T3 (mouse embryonic fibroblast)	Euk	Plunge	FIB-SEM		7	15		https://bionumbers.hms.harvard.edu/files/Sizes%20of%20various%20cells.pdf	1.20	cone	179	10.1016/j.cell.2024.07.020
NIH/3T3 (mouse embryonic fibroblast)	Euk	Plunge	FIB-SEM		7	15		https://bionumbers.hms.harvard.edu/files/Sizes%20of%20various%20cells.pdf	1.20	cone	143	10.1016/j.jsb.2020.107633
Ostreococcus tauri	Euk	Plunge	none		0.65	1.5		17710148	12.00	capsule	180	10.1371/journal.pone.0000749
Ostreococcus tauri	Euk	Plunge	none		0.65	1.5		17710148	12.00	capsule	181	10.1091/mbc.E17-07-0449
P19 (mouse embryonic cancer)	Euk	Plunge	Ultramicrotome		7	20		20163207	0.90	cone	147	10.1016/j.jsb.2007.10.008
P19 (mouse embryonic cancer)	Euk	Plunge	FIB-SEM		7	20		20163207	0.90	cone	104	10.1038/s41592-021-01275-4
PC12 (rat adrenal gland pheochromocytoma)	Euk	Plunge	none		7	12		36602900	1.50	cone	182	10.1002/1873-3468.13316
PC3 (human prostate cancer)	Euk	Plunge	FIB-SEM		7	20		26241348	0.90	cone	183	10.1038/s41594-023-01118-0
Phaeodactylum tricornutum	Euk	Plunge	FIB-SEM		3	20		30254372	0.90	capsule	184	10.1016/j.cell.2024.09.013
Pichia pastoris	Euk	Plunge	FIB-SEM		5	5		28559166	3.60	sphere	185	10.1016/j.jsb.2017.05.011

Plasmodium berghei (sporozite)	Euk	Plunge	none		1	11		28108531	1.64	capsule	186	10.1111/j.1462-5822.2009.01399.x
Plasmodium berghei (sporozite)	Euk	Plunge	none		1	11		28108531	1.64	capsule	187	10.1186/1757-5036-3-6
Plasmodium berghei (sporozoites)	Euk	Plunge	none		1	11		28108531	1.64	capsule	188	10.1111/j.1462-5822.2012.01836.x
Plasmodium falciparum (merozoites)	Euk	Plunge	none		1	1.5		34286696	12.00	capsule	189	10.1111/cmi.12132
Pleurochrysis carterae (coccoliths)	Euk	Plunge	none		7.5	7.5		32018012	2.40	sphere	190	10.1016/j.jsb.2020.107476
Pleurochrysis carterae (coccoliths)	Euk	Plunge	none		7.5	7.5		32018012	2.40	sphere	191	10.1016/j.jsb.2019.08.007
PtK-1 (rat kangaroo epithelial kidney)	Euk	Plunge	FIB-SEM		7	30		36539423	0.60	cone	192	10.1038/s41467-022-35409-9
PtK-2 (potoroo epithelial kidney)	Euk	Plunge	none		3	37		39308425	0.49	cone	193	
PtK-2 (potoroo epithelial kidney)	Euk	Plunge	none		3	37		39308425	0.49	cone	194	10.1016/j.jsb.2011.11.018
PtK-2 (potoroo epithelial kidney)	Euk	Plunge	none		3	37		39308425	0.49	cone	193	10.1242/jcs.262163
Rat liver tissue	Euk	HPF	Ultramicrotome	20% dextran	50	1000	1000	16343943	0.02	rectangle	195	10.1016/j.jsb.2005.10.004
REF52 (rat embryo fibroblast)	Euk	Plunge	none		7	45		32444491	0.40	cone	116	10.1016/j.jsb.2011.08.012
Rhesus macaque fibroblasts	Euk	Plunge	none		7	40		29078993	0.45	cone	196	10.1016/j.jsb.2017.10.009
Rod outer segment (mouse)	Euk	Plunge	none		2	25	2	32176260	0.72	rectangle	197	10.1016/j.cell.2012.10.038
Saccharomyces cerevisiae	Euk	Plunge	FIB-SEM		5	5		36707648	3.60	sphere	105	10.7554/eLife.7050610.7554/eLife.70506.sa010.7554/eLife.70506.sa110.7554/eLife.70506.sa2
Saccharomyces cerevisiae	Euk	Plunge	FIB-SEM		5	5		36707648	3.60	sphere	198	10.1016/j.str.2020.07.017
Saccharomyces cerevisiae	Euk	Plunge	Plasma FIB-SEM	glycerol	5	5		36707648	3.60	sphere	129	10.1038/s41467-023-36372-9
Saccharomyces cerevisiae	Euk	HPF	FIB-SEM		5	5		36707648	3.60	sphere	43	10.1038/s41467-022-29501-3
Saccharomyces cerevisiae	Euk	Plunge	FIB-SEM		5	5		36707648	3.60	sphere	99	10.1016/j.jsb.2024.108163

Saccharomyces cerevisiae	Euk	Plunge	FIB-SEM	15% high molecular weight dextran	5	5		36707648	3.60	sphere	199	10.1016/j.devcel.2019.09.019
Saccharomyces cerevisiae	Euk	Plunge	Ultramicrotome	50% dextran	5	5		36707648	3.60	sphere	200	10.1083/jcb.201809088
Saccharomyces cerevisiae	Euk	Plunge	Ultramicrotome		5	5		36707648	3.60	sphere	72	10.1016/j.jsb.2010.02.011
Saccharomyces cerevisiae	Euk	Plunge	FIB-SEM		5	5		36707648	3.60	sphere	201	10.1016/j.cell.2021.12.015
Saccharomyces cerevisiae	Euk	HPF	Ultramicrotome	20% dextran	5	5		36707648	3.60	sphere	202	10.1091/mbc.E11-10-0850
Saccharomyces cerevisiae	Euk	Plunge	FIB-SEM		5	5		36707648	3.60	sphere	203	10.1016/j.molcel.2020.10.030
Saccharomyces cerevisiae	Euk	Plunge	FIB-SEM		5	5		36707648	3.60	sphere	204	10.1016/j.devcel.2019.10.018
Saccharomyces cerevisiae	Euk	HPF	Ultramicrotome		5	5		36707648	3.60	sphere	205	10.1016/j.jsb.2010.07.004
Saccharomyces cerevisiae	Euk	HPF	Ultramicrotome	20% dextran	5	5		36707648	3.60	sphere	206	10.1016/j.jsb.2009.10.001
Saccharomyces cerevisiae	Euk	Plunge	FIB-SEM		5	5		36707648	3.60	sphere	179	10.1016/j.cell.2024.07.020
Saccharomyces cerevisiae	Euk	Plunge	FIB-SEM		5	5		36707648	3.60	sphere	207	10.1083/jcb.202205053
Saccharomyces cerevisiae	Euk	HPF	Ultramicrotome		5	5		36707648	3.60	sphere	208	10.1016/j.jsb.2013.05.016
Saccharomyces cerevisiae	Euk	HPF	Ultramicrotome	20% dextran	5	5		36707648	3.60	sphere	209	10.1111/j.1600-0854.2008.00789.x
Saccharomyces cerevisiae	Euk	HPF	Ultramicrotome	20% dextran	5	5		36707648	3.60	sphere	210	10.1016/j.jsb.2013.11.005
Saccharomyces cerevisiae	Euk	Plunge	FIB-SEM		5	5		36707648	3.60	sphere	211	10.1016/j.cell.2024.04.026
Sf9 (insect)	Euk	Plunge	FIB-SEM		7	16		23884632	1.13	cone	50	10.1016/j.jsb.2016.02.013
Sperm (Ascaris)	Euk	Plunge	FIB-SEM		15	15		2910878	1.20	capsule	50	10.1016/j.jsb.2016.02.013
Sperm (horse)	Euk	Plunge	FIB-SEM		3	6		doi.org/10.1016/j.jevs.2015.01.005	3.00	cone	212	10.15252/embj.2020107410
Sperm (horse)	Euk	Plunge	none		3	6		doi.org/10.1016/j.jevs.2015.01.005	3.00	cone	213	10.3389/fcell.2021.772254
Sperm (human)	Euk	Plunge	none		2.8	4.4		20852650	4.09	cone	214	10.3402/jev.v4.28680
Sperm (human)	Euk	Plunge	FIB-SEM		2.8	4.4		20852650	4.09	cone	215	10.1038/s41594-022-00861-0

Sperm (human)	Euk	Plunge	none		2.8	4.4		20852650	4.09	cone	216	10.1002/1873-3468.13379
Sperm (mouse)	Euk	Plunge	FIB-SEM		4	7		24338943	2.57	cone	212	10.15252/embj.2020107410
Sperm (mouse)	Euk	Plunge	FIB-SEM		4	7		24338943	2.57	cone	215	10.1038/s41594-022-00861-0
Sperm (mouse)	Euk	Plunge	FIB-SEM		4	7		24338943	2.57	cone	217	10.1038/s41421-023-00606-3
Sperm (pig)	Euk	Plunge	FIB-SEM		4.5	9		17555808	2.00	cone	212	10.15252/embj.2020107410
Sperm (sea urchin)	Euk	Plunge	none		1.3	3.6		31337311	5.00	cone	109	10.1038/ncb2939
Spinach leaf	Euk	HPF	Ultramicrotome	1-hexadecene, dextran	20	100	30	34174447	0.18	rectangle	166	10.1016/j.jsb.2021.107763
Sum159 (human breast cancer)	Euk	Plunge	FIB-SEM		7	40		34951584	0.45	cone	105	10.7554/eLife.7050610.7554/eLife.70506.sa010.7554/eLife.70506.sa110.7554/eLife.70506.sa2
Thalassiosira pseudonana	Euk	Plunge	FIB-SEM		9	9		19269330	2.00	sphere	184	10.1016/j.cell.2024.09.013
Theileria annulata	Euk	Plunge	none		2	10		22891986	1.80	capsule	218	10.1111/cmi.12006
TIFF (human telomerase foreskin fibroblasts)	Euk	HPF	Cryo-FIB lift-out, FIB-SEM	dextran, sucrose, polyvinylpyrrolidone, BSA	7	20		38506714	0.90	cone	219	10.1083/jcb.202309125
Toxoplasma gondii	Euk	Plunge	none		2	6		9564564	3.00	capsule	220	10.1073/pnas.2111661119
Toxoplasma gondii	Euk	Plunge	none		2	6		9564564	3.00	capsule	221	
Toxoplasma gondii	Euk	Plunge	FIB-SEM		2	6		9564564	3.00	capsule	179	10.1016/j.cell.2024.07.020
Toxoplasma gondii	Euk	Plunge	none		2	6		9564564	3.00	capsule	222	10.15252/embj.2022111158
Trypanosoma brucei	Euk	Plunge	none		3	25		15525435	0.72	capsule	223	10.1016/j.jsb.2012.01.009
Trypanosoma brucei	Euk	HPF	Ultramicrotome	20% dextran, 0.1% sucrose	3	25		15525435	0.72	capsule	223	10.1016/j.jsb.2012.01.009
U2OS (human osteosarcoma)	Euk	Plunge	FIB-SEM		7	30		24764273	0.60	cone	98	10.1038/s41596-020-0320-x
U2OS (human osteosarcoma)	Euk	Plunge	none		7	30		24764273	0.60	cone	224	10.1016/j.str.2012.03.025
U2OS (human osteosarcoma)	Euk	Plunge	none		7	30		24764273	0.60	cone	40	10.1111/cmi.12310

U2OS (human osteosarcoma)	Euk	Plunge	FIB-SEM		7	30		24764273	0.60	cone	[225]	10.1083/jcb.202206143
U2OS (U2OS-IRE1a-mNG) (human osteosarcoma)	Euk	Plunge	none		7	30		24764273	0.60	cone	[157]	10.1126/science.abh2474
VeroE6 (monkey kidney)	Euk	Plunge	Ultramicrotome		7	17.5		31127400	1.03	cone	[15]	10.1016/j.jsb.2012.12.004
WI38 (human lung fibroblasts)	Euk	Plunge	none		7	10	90	37268154	1.80	rectangular based pyramid	[226]	10.1016/j.jsb.2023.107982
WI38 (human lung fibroblasts)	Euk	Plunge	none		7	10	90	37268154	1.80	rectangular based pyramid	[227]	10.1016/j.yjsbx.2018.100002

Supplementary Table 2: Distribution of Cellular Domains by Web of Science Search Term

Domain	Percentage of Studies (%)		
	'cryogenic electron tomography'	*'electron cryotomography'*	*'cryo-electron tomography'*
Arch	7	2	1
Bact	57	79	19
Euk	36	19	80

Supplementary References:

1. Yelton, A. P. *et al.* Comparative genomics in acid mine drainage biofilm communities reveals metabolic and structural differentiation of co-occurring archaea. *BMC Genomics* **14**, 1–15 (2013).
2. Comolli, L. R., Baker, B. J., Downing, K. H., Siegerist, C. E. & Banfield, J. F. Three-dimensional analysis of the structure and ecology of a novel, ultra-small archaeon. *ISME J.* **3**, 159–167 (2009).
3. Briegel, A. *et al.* Structural conservation of chemotaxis machinery across Archaea and Bacteria. *Environ. Microbiol. Rep.* **7**, 414–419 (2015).
4. Burns, D. G. *et al.* Haloquadratum walsbyi gen. nov., sp. nov., the square haloarchaeon of Walsby, isolated from saltern crystallizers in Australia and Spain. *Int. J. Syst. Evol. Microbiol.* **57**, 387–392 (2007).
5. Wang, H. *et al.* Hierarchical organization and assembly of the archaeal cell sheath from an amyloid-like protein. *Nat. Commun.* **14**, 1–11 (2023).
6. Nickell, S., Hegerl, R., Baumeister, W. & Rachel, R. Pyrodictium cannulae enter the periplasmic space but do not enter the cytoplasm, as revealed by cryo-electron tomography. *J. Struct. Biol.* **141**, 34–42 (2003).
7. Dobro, M. J. *et al.* Electron cryotomography of ESCRT assemblies and dividing Sulfolobus cells suggests that spiraling filaments are involved in membrane scission. *Mol. Biol. Cell* **24**, 2319–2327 (2013).
8. Fu, C. Y. *et al.* In vivo assembly of an archaeal virus studied with whole-cell electron cryotomography. *Structure* **18**, 1579–1586 (2010).
9. Briegel, A. *et al.* Morphology of the archaellar motor and associated cytoplasmic cone in Thermococcus kodakaraensis . *EMBO Rep.* **18**, 1660–1670 (2017).
10. Kaplan, M. *et al.* Loss of the bacterial flagellar motor switch complex upon cell lysis. *MBio* **12**, e00298-21 (2021).
11. Chen, S. *et al.* Structural diversity of bacterial flagellar motors. *EMBO J.* **30**, 2972–2981 (2011).
12. Xu, M. *et al.* De Novo Structural Pattern Mining in Cellular Electron Cryotomograms. *Structure* **27**, 679-691.e14 (2019).
13. Tocheva, E. I. *et al.* Polyphosphate storage during sporulation in the Gram-negative bacterium Acetonema longum. *J. Bacteriol.* **195**, 3940–3946 (2013).
14. Tocheva, E. I. *et al.* Peptidoglycan remodeling and conversion of an inner membrane into an outer membrane during sporulation. *Cell* **146**, 799–812 (2011).
15. Faas, F. G. A. *et al.* Localization of fluorescently labeled structures in frozen-hydrated samples using integrated light electron microscopy. *J. Struct. Biol.* **181**, 283–290 (2013).
16. Nicolas, W. J., Ghosal, D., Tocheva, E. I., Meyerowitz, E. M. & Jensen, G. J. Structure of the bacterial cellulose ribbon and its assembly-guiding cytoskeleton by electron cryotomography. *J. Bacteriol.* **203**, e00371-20 (2021).
17. Böck, D. *et al.* In situ architecture, function, and evolution of a contractile injection system. *Science.* **717**, 713–717 (2017).

18. Weiss, G. L., Kieninger, A. K., Maldener, I., Forchhammer, K. & Pilhofer, M. Structure and Function of a Bacterial Gap Junction Analog. *Cell* **178**, 374-384.e15 (2019).
19. Dobro, M. J. *et al.* Uncharacterized bacterial structures revealed by electron cryotomography. *J. Bacteriol.* **199**, 1–14 (2017).
20. Tocheva, E. I. *et al.* Peptidoglycan transformations during Bacillus subtilis sporulation. *Mol. Microbiol.* **88**, 673–686 (2013).
21. Lopez-Garrido, J. *et al.* Chromosome Translocation Inflates Bacillus Forespores and Impacts Cellular Morphology. *Cell* **172**, 758-770.e14 (2018).
22. Farley, M. M., Tu, J., Kearns, D. B., Molineux, I. J. & Liu, J. Ultrastructural analysis of bacteriophage Φ29 during infection of Bacillus subtilis. *J Struct Biol* **197**, 163–171 (2018).
23. Kaplan, M. *et al.* Bdellovibrio predation cycle characterized at nanometre-scale resolution with cryo-electron tomography. *Nat. Microbiol.* **8**, 1267–1279 (2023).
24. Chreifi, G., Chen, S., Metskas, L. A., Kaplan, M. & Jensen, G. J. Rapid tilt-series acquisition for electron cryotomography. *J. Struct. Biol.* **205**, 163–169 (2019).
25. Yao, Q. *et al.* Short FtsZ filaments can drive asymmetric cell envelope constriction at the onset of bacterial cytokinesis. *EMBO J.* **36**, 1577–1589 (2017).
26. Kudryashev, M., Cyrklaff, M., Wallich, R., Baumeister, W. & Frischknecht, F. Distinct in situ structures of the Borrelia flagellar motor. *J. Struct. Biol.* **169**, 54–61 (2010).
27. Kudryashev, M. *et al.* Evidence of direct cell-cell fusion in Borrelia by cryogenic electron tomography. *Cell. Microbiol.* **13**, 731–741 (2011).
28. Genthe, E. *et al.* PickYOLO: Fast deep learning particle detector for annotation of cryo electron tomograms. *J. Struct. Biol.* **215**, 107990 (2023).
29. Kaplan, M. *et al.* In situ imaging of bacterial outer membrane projections and associated protein complexes using electron cryo-tomography. *Elife* **10**, 1–24 (2021).
30. Müller, A. *et al.* Ultrastructure and complex polar architecture of the human pathogen Campylobacter jejuni. *Microbiologyopen* **3**, 702–710 (2014).
31. Dahlberg, P. D. *et al.* Cryogenic single-molecule fluorescence annotations for electron tomography reveal in situ organization of key proteins in Caulobacter. *Proc. Natl. Acad. Sci. U. S. A.* **117**, 13937–13944 (2020).
32. Kudryashev, M., Stahlberg, H. & Castaño-Díez, D. Assessing the benefits of focal pair cryo-electron tomography. *J. Struct. Biol.* **178**, 88–97 (2012).
33. Dahlberg, P. D., Perez, D., Hecksel, C. W., Chiu, W. & Moerner, W. E. Metallic Support Films Reduce Optical Heating in Cryogenic Correlative Light and Electron Tomography. *J. Struct. Biol.* **214**, 107901 (2022).
34. Perez, D. *et al.* Identification and demonstration of roGFP2 as an environmental sensor for cryogenic correlative light and electron microscopy. *J. Struct. Biol.* **214**, 107881 (2022).

35. Yoniles, J. *et al.* Time-resolved cryogenic electron tomography for the study of transient cellular processes. *Mol. Biol. Cell* **35**, 1–7 (2024).
36. Gan, L., Chen, S. & Jensen, G. J. Molecular organization of Gram-negative peptidoglycan. *Proc. Natl. Acad. Sci. U. S. A.* **105**, 18953–18957 (2008).
37. Li, Z., Trimble, M. J., Brun, Y. V. & Jensen, G. J. The structure of FtsZ filaments in vivo suggests a force-generating role in cell division. *EMBO J.* **26**, 4694–4708 (2007).
38. von Kügelgen, A. *et al.* In Situ Structure of an Intact Lipopolysaccharide-Bound Bacterial Surface Layer. *Cell* **180**, 348-358.e15 (2020).
39. Comolli, L. R. & Downing, K. H. Dose tolerance at helium and nitrogen temperatures for whole cell electron tomography. *J. Struct. Biol.* **152**, 149–156 (2005).
40. Nans, A., Saibil, H. R. & Hayward, R. D. Pathogen-host reorganization during Chlamydia invasion revealed by cryo-electron tomography. *Cell. Microbiol.* **16**, 1457–1472 (2014).
41. Sexton, D. L., Burgold, S., Schertel, A. & Tocheva, E. I. Super-resolution confocal cryo-CLEM with cryo-FIB milling for in situ imaging of Deinococcus radiodurans. *Curr. Res. Struct. Biol.* **4**, 1–9 (2022).
42. Tacke, S. *et al.* A streamlined workflow for automated cryo focused ion beam milling. *J. Struct. Biol.* **213**, 107743 (2021).
43. Kelley, K. *et al.* Waffle Method: A general and flexible approach for improving throughput in FIB-milling. *Nat. Commun.* **13**, 1–13 (2022).
44. Szwedziak, P., Wang, Q., Freund, S. M. & Löwe, J. FtsA forms actin-like protofilaments. *EMBO J.* **31**, 2249–2260 (2012).
45. Wang, Q., Mercogliano, C. P. & Löwe, J. A ferritin-based label for cellular electron cryotomography. *Structure* **19**, 147–154 (2011).
46. Li, S. *et al.* High-vacuum optical platform for cryo-CLEM (HOPE): A new solution for non-integrated multiscale correlative light and electron microscopy. *J. Struct. Biol.* **201**, 63–75 (2018).
47. Pilhofer, M., Ladinsky, M. S., McDowall, A. W., Petroni, G. & Jensen, G. J. Microtubules in Bacteria: Ancient tubulins build a five-protofilament homolog of the eukaryotic cytoskeleton. *PLoS Biol.* **9**, e1001213 (2011).
48. Hale, V. L., Hooker, J., Russo, C. J. & Löwe, J. Honeycomb gold specimen supports enabling orthogonal focussed ion beam-milling of elongated cells for cryo-ET. *J. Struct. Biol.* **216**, 108097 (2024).
49. Khavnekar, S. *et al.* Multishot tomography for high-resolution in situ subtomogram averaging. *J. Struct. Biol.* **215**, 107911 (2023).
50. Zhang, J., Ji, G., Huang, X., Xu, W. & Sun, F. An improved cryo-FIB method for fabrication of frozen hydrated lamella. *J. Struct. Biol.* **194**, 218–223 (2016).
51. Rapisarda, C. *et al.* In situ and high-resolution cryo- EM structure of a bacterial type VI secretion system membrane complex . *EMBO J.* **38**, 1–18 (2019).

52. Wang, K., Strunk, K., Zhao, G., Gray, J. L. & Zhang, P. 3D structure determination of native mammalian cells using cryo-FIB and cryo-electron tomography. *J. Struct. Biol.* **180**, 318–326 (2012).
53. Fu, X. *et al.* Controlled bacterial lysis for electron tomography of native cell membranes. *Structure* **22**, 1875–1882 (2014).
54. Navarro, P. P. *et al.* Cell wall synthesis and remodelling dynamics determine division site architecture and cell shape in Escherichia coli. *Nat. Microbiol.* **7**, 1621–1634 (2022).
55. Boge, L. *et al.* Peptide-Loaded Cubosomes Functioning as an Antimicrobial Unit against Escherichia coli. *ACS Appl. Mater. Interfaces* **11**, 21314–21322 (2019).
56. Ruhe, Z. C. *et al.* Programmed Secretion Arrest and Receptor-Triggered Toxin Export during Antibacterial Contact-Dependent Growth Inhibition. *Cell* **175**, 921-933.e14 (2018).
57. Khursigara, C. M. *et al.* Lateral density of receptor arrays in the membrane plane influences sensitivity of the E. coli chemotaxis response. *EMBO J.* **30**, 1719–1729 (2011).
58. Howley, E., Mangus, A., Williams, D. & Torres, C. I. Intracytoplasmic membranes develop in Geobacter sulfurreducens under thermodynamically limiting conditions. *npj Biofilms Microbiomes* **18**, 1–8 (2023).
59. Iancu, C. V *et al.* Organization, Structure, and Assembly of α-Carboxysomes Determined by Electron Cryotomography of Intact Cells. *J. Mol. Biol.* **396**, 105–117 (2010).
60. Tachiyama, S. *et al.* The flagellar motor protein FliL forms a scaffold of circumferentially positioned rings required for stator activation. *Proc. Natl. Acad. Sci. U. S. A.* **119**, 1–9 (2022).
61. Chang, Y. W., Shaffer, C. L., Rettberg, L. A., Ghosal, D. & Jensen, G. J. In Vivo Structures of the Helicobacter pylori cag Type IV Secretion System. *Cell Rep.* **23**, 673–681 (2018).
62. Ghosal, D., Chang, Y., Jeong, K. C., Vogel, J. P. & Jensen, G. J. In situ structure of the Legionella Dot/Icm type IV secretion system by electron cryotomography . *EMBO Rep.* **18**, 726–732 (2017).
63. Ghosal, D. *et al.* Molecular architecture, polar targeting and biogenesis of the Legionella Dot/Icm T4SS. *Nat. Microbiol.* **4**, 1173–1182 (2019).
64. Song, K., Comolli, L. R. & Horowitz, M. Removing high contrast artifacts via digital inpainting in cryo-electron tomography: An application of compressed sensing. *J. Struct. Biol.* **178**, 108–120 (2012).
65. Fukuda, Y., Laugks, U., Lučić, V., Baumeister, W. & Danev, R. Electron cryotomography of vitrified cells with a Volta phase plate. *J. Struct. Biol.* **190**, 143–154 (2015).
66. Cornejo, E., Subramanian, P., Li, Z., Jensen, G. J. & Komeili, A. Dynamic remodeling of the magnetosome membrane is triggered by the initiation of biomineralization. *MBio* **7**, e01898-15 (2016).
67. Abreu, F. *et al.* Cryo-electron tomography of the magnetotactic vibrio Magnetovibrio blakemorei: Insights into the biomineralization of prismatic magnetosomes. *J. Struct. Biol.* **181**, 162–168 (2013).
68. Iancu, C. V., Wright, E. R., Heymann, J. B. & Jensen, G. J. A comparison of liquid nitrogen and liquid helium as cryogens for

electron cryotomography. *J. Struct. Biol.* **153**, 231–240 (2006).
69. Iancu, C. V. *et al.* A 'flip-flop' rotation stage for routine dual-axis electron cryotomography. *J. Struct. Biol.* **151**, 288–297 (2005).
70. Ortega, D. R. *et al.* Repurposing a chemosensory macromolecular machine. *Nat. Commun.* **11**, 1–13 (2020).
71. Tavormina, P. L. *et al.* Starvation and recovery in the deep-sea methanotroph Methyloprofundus sedimenti. *Mol. Microbiol.* **103**, 242–252 (2017).
72. Rigort, A. *et al.* Micromachining tools and correlative approaches for cellular cryo-electron tomography. *J. Struct. Biol.* **172**, 169–179 (2010).
73. Yu, Z. & Frangakis, A. S. M-free: Scoring the reference bias in sub-tomogram averaging and template matching. *J. Struct. Biol.* **187**, 10–19 (2014).
74. Henderson, G. P. & Jensen, G. J. Three-dimensional structure of Mycoplasma pneumoniae's attachment organelle and a model for its role in gliding motility. *Mol. Microbiol.* **60**, 376–385 (2006).
75. Seybert, A., Herrmann, R. & Frangakis, A. S. Structural analysis of Mycoplasma pneumoniae by cryo-electron tomography. *J. Struct. Biol.* **156**, 342–354 (2006).
76. Chang, Y. W. *et al.* Correlated cryogenic photoactivated localization microscopy and cryo-electron tomography. *Nat. Methods* **11**, 737–739 (2014).
77. Chang, Y., Rettberg, L. A., Ortega, D. R. & Jensen, G. J. In vivo structures of an intact type VI secretion system revealed by electron cryotomography . *EMBO Rep.* **18**, 1090–1099 (2017).
78. Pilhofer, M. *et al.* Architecture and host interface of environmental chlamydiae revealed by electron cryotomography. *Environ. Microbiol.* **16**, 417–429 (2014).
79. Liu, X. *et al.* Structural changes in a marine podovirus associated with release of its genome into Prochlorococcus. *Nat. Struct. Mol. Biol.* **17**, 830–836 (2010).
80. Kaplan, M., Yao, Q. & Jensen, G. J. Structure and Assembly of the Proteus mirabilis Flagellar Motor by Cryo-Electron Tomography. *Int. J. Mol. Sci.* **24**, 1–8 (2023).
81. Delgado, L., Martínez, G., López-Iglesias, C. & Mercadé, E. Cryo-electron tomography of plunge-frozen whole bacteria and vitreous sections to analyze the recently described bacterial cytoplasmic structure, the Stack. *J. Struct. Biol.* **189**, 220–229 (2015).
82. Beeby, M., Cho, M., Stubbe, J. & Jensen, G. J. Growth and localization of polyhydroxybutyrate granules in ralstonia eutropha. *J. Bacteriol.* **194**, 1092–1099 (2012).
83. Danita, C., Chiu, W. & Galaz-Montoya, J. G. Efficient manual annotation of cryogenic electron tomograms using IMOD. *STAR Protoc.* **3**, 1–75 (2022).
84. Konorty, M., Kahana, N., Linaroudis, A., Minsky, A. & Medalia, O. Structural analysis of photosynthetic membranes by cryo-

electron tomography of intact Rhodopseudomonas viridis cells. *J. Struct. Biol.* **161**, 393–400 (2008).
85. Hu, B., Lara-Tejero, M., Kong, Q., Galán, J. E. & Liu, J. In Situ Molecular Architecture of the Salmonella Type III Secretion Machine. *Cell* **168**, 1065-1074.e10 (2017).
86. Subramanian, P., Pirbadian, S., El-Naggar, M. Y. & Jensen, G. J. Ultrastructure of Shewanella oneidensis MR-1 nanowires revealed by electron cryotomography. *Proc. Natl. Acad. Sci. U. S. A.* **115**, E3246–E3255 (2018).
87. Gupta, T. K. *et al.* Structural basis for VIPP1 oligomerization and maintenance of thylakoid membrane integrity. *Cell* **184**, 3643–3659 (2021).
88. Osman, S. *et al.* Tetrasphaera remsis sp. nov., isolated from the regenerative enclosed life support module simulator (REMS) air system. *Int. J. Syst. Evol. Microbiol.* **57**, 2749–2753 (2007).
89. Izard, J., Hsieh, C. E., Limberger, R. J., Mannella, C. A. & Marko, M. Native cellular architecture of Treponema denticola revealed by cryo-electron tomography. *J. Struct. Biol.* **163**, 10–17 (2008).
90. Kurniyati, K., Liu, J., Zhang, J. R., Min, Y. & Li, C. A pleiotropic role of FlaG in regulating the cell morphogenesis and flagellar homeostasis at the cell poles of Treponema denticola. *Cell. Microbiol.* **21**, 1–12 (2019).
91. Murphy, G. E., Leadbetter, J. R. & Jensen, G. J. In situ structure of the complete Treponema primitia flagellar motor. *Nature* **442**, 1062–1064 (2006).
92. Depelteau, J. S. *et al.* UVC inactivation of pathogenic samples suitable for cryo-EM analysis. *Commun. Biol.* **5**, 1–8 (2022).
93. Basler, M., Pilhofer, M., Henderson, G. P., Jensen, G. J. & Mekalanos, J. J. Type VI secretion requires a dynamic contractile phage tail-like structure. *Nature* **483**, 182–186 (2012).
94. Berger, C. *et al.* Structure of the Yersinia injectisome in intracellular host cell phagosomes revealed by cryo FIB electron tomography. *J. Struct. Biol.* **213**, (2021).
95. Serwas, D. *et al.* Mechanistic insights into actin force generation during vesicle formation from cryo-electron tomography. *Dev. Cell* **57**, 1132-1145.e5 (2022).
96. Wolff, G. *et al.* Mind the gap: Micro-expansion joints drastically decrease the bending of FIB-milled cryo-lamellae. *J. Struct. Biol.* **208**, 107389 (2019).
97. Takvorian, P. M. *et al.* An Ultrastructural Study of the Extruded Polar Tube of Anncaliia algerae (Microsporidia). *J. Eukaryot. Microbiol.* **67**, 28–44 (2020).
98. Wagner, F. R. *et al.* Preparing samples from whole cells using focused-ion-beam milling for cryo-electron tomography. *Nat. Protoc.* **15**, 2041–2070 (2020).
99. Inacio Costa-Filho, J., Theveny, L., De Sautu, M. & Kirchhausen, T. CryoSamba: self-supervised deep volumetric denoising for cryo-1 electron tomography data 2 3 4. *J. Struct. Biol.* **217**, 108163 (2025).
100. Harapin, J. *et al.* Structural analysis of multicellular organisms with cryo-electron tomography. *Nat. Methods* **12**, 634–636 (2015).

101. Schiøtz, O. H. *et al.* Serial Lift-Out: sampling the molecular anatomy of whole organisms. *Nat. Methods* **21**, 1684–1692 (2024).
102. Mahamid, J. *et al.* A focused ion beam milling and lift-out approach for site-specific preparation of frozen-hydrated lamellas from multicellular organisms. *J. Struct. Biol.* **192**, 262–269 (2015).
103. Zhu, H., Li, M., Li, M., Li, X. & Ou, G. Cryo-electron tomography elucidates annular intraluminal configurations in Caenorhabditis elegans microtubules. *Biol. Cell* **116**, 1–12 (2024).
104. Moebel, E. *et al.* Deep learning improves macromolecule identification in 3D cellular cryo-electron tomograms. *Nat. Methods* **18**, 1386–1394 (2021).
105. Klumpe, S. *et al.* A Modular Platform for Automated Cryo-FIB Workflows. *Elife* **10**, 1–29 (2021).
106. Bui, K. H., Sakakibara, H., Movassagh, T., Oiwa, K. & Ishikawa, T. Asymmetry of inner dynein arms and inter-doublet links in Chlamydomonas flagella. *J. Cell Biol.* **186**, 437–446 (2009).
107. Schaffer, M. *et al.* Optimized cryo-focused ion beam sample preparation aimed at in situ structural studies of membrane proteins. *J. Struct. Biol.* **197**, 73–82 (2017).
108. Jordan, M. A., Diener, D. R., Stepanek, L. & Pigino, G. The cryo-EM structure of intraflagellar transport trains reveals how dynein is inactivated to ensure unidirectional anterograde movement in cilia. *Nat. Cell Biol.* **20**, 1250–1255 (2018).
109. Lin, J., Okada, K., Raytchev, M., Smith, M. C. & Nicastro, D. Structural mechanism of the dynein power stroke. *Nat. Cell Biol.* **16**, 479–485 (2014).
110. Lacey, S. E., Foster, H. E. & Pigino, G. The molecular structure of IFT-A and IFT-B in anterograde intraflagellar transport trains. *Nat. Struct. Mol. Biol.* **30**, 584–593 (2023).
111. Klena, N. *et al.* Architecture of the centriole cartwheel-containing region revealed by cryo-electron tomography. *EMBO J.* **39**, 1–17 (2020).
112. Craig, E. W. *et al.* The elusive actin cytoskeleton of a green alga expressing both conventional and divergent actins. *Mol. Biol. Cell* **30**, 2827–2837 (2019).
113. Lin, C., Zhang, L., Zhang, Z., Jiang, Y. & Li, X. Locating cellular contents during cryoFIB milling using cellular secondary-electron imaging. *J. Struct. Biol.* **215**, 108005 (2023).
114. Bouchet-Marquis, C. *et al.* Visualization of cell microtubules in their native state. *Biol. Cell* **99**, 45–53 (2007).
115. Elad, N., Abramovitch, S., Sabanay, H. & Medalia, O. Microtubule organization in the final stages of cytokinesis as revealed by cryo-electron tomography. *J. Cell Sci.* **124**, 207–215 (2011).
116. Rigort, A. *et al.* Automated segmentation of electron tomograms for a quantitative description of actin filament networks. *J. Struct. Biol.* **177**, 135–144 (2012).
117. Jasnin, M., Ecke, M., Baumeister, W. & Gerisch, G. Actin Organization in Cells Responding to a Perforated Surface, Revealed by Live Imaging and Cryo-Electron Tomography. *Structure* **24**, 1031–1043 (2016).

118. Jasnin, M. *et al.* The Architecture of Traveling Actin Waves Revealed by Cryo-Electron Tomography. *Structure* **27**, 1211-1223.e5 (2019).
119. Leforestier, A. *et al.* Imaging Drosophila brain by combining cryo-soft X-ray microscopy of thick vitreous sections and cryo-electron microscopy of ultrathin vitreous sections. *J. Struct. Biol.* **188**, 177–182 (2014).
120. Ventura Santos, C., Rogers, S. L. & Carter, A. P. CryoET shows cofilactin filaments inside the microtubule lumen . *EMBO Rep.* **24**, 1–13 (2023).
121. Paul, D. M. *et al.* In situ cryo-electron tomography reveals filamentous actin within the microtubule lumen. *J. Cell Biol.* **219**, e201911154 (2020).
122. Liu, B. *et al.* Three-dimensional super-resolution protein localization correlated with vitrified cellular context. *Sci. Rep.* **5**, 1–11 (2015).
123. Zhao, D. Y. *et al.* Autophagy preferentially degrades non-fibrillar polyQ aggregates. *Mol. Cell* **84**, 1980-1994.e8 (2024).
124. Girard, J. *et al.* In situ fate of Chikungunya virus replication organelles. *J. Virol.* **98**, 1–20 (2024).
125. Silvester, E. *et al.* DNA origami signposts for identifying proteins on cell membranes by electron cryotomography. *Cell* **184**, 1110-1121.e16 (2021).
126. Watanabe, R. *et al.* The In Situ Structure of Parkinson’s Disease-Linked LRRK2. *Cell* **182**, 1508-1518.e16 (2020).
127. Fry, M. Y. *et al.* In situ architecture of Opa1-dependent mitochondrial cristae remodeling. *EMBO J.* **43**, 391–413 (2024).
128. Watanabe, R. *et al.* Intracellular Ebola virus nucleocapsid assembly revealed by in situ cryo-electron tomography. *Cell* **187**, 5587-5603.e19 (2024).
129. Berger, C. *et al.* Plasma FIB milling for the determination of structures in situ. *Nat. Commun.* **14**, 1–12 (2023).
130. Huebinger, J. *et al.* Direct Measurement of Water States in Cryopreserved Cells Reveals Tolerance toward Ice Crystallization. *Biophys. J.* **110**, 840–849 (2016).
131. Carter, S. D. *et al.* Distinguishing signal from autofluorescence in cryogenic correlated light and electron microscopy of mammalian cells. *J. Struct. Biol.* **201**, 15–25 (2018).
132. Yang, J. E., Larson, M. R., Sibert, B. S., Shrum, S. & Wright, E. R. CorRelator: Interactive software for real-time high precision cryo-correlative light and electron microscopy. *J. Struct. Biol.* **213**, 107709 (2021).
133. Carter, S. D., Mamede, J. I., Hope, T. J. & Jensen, G. J. Correlated cryogenic fluorescence microscopy and electron cryo-tomography shows that exogenous TRIM5a can form hexagonal lattices or autophagy aggregates in vivo. *Proc. Natl. Acad. Sci. U. S. A.* **117**, 29702–29711 (2020).
134. Li, W. *et al.* Integrated multimodality microscope for accurate and efficient target-guided cryo-lamellae preparation. *Nat. Methods* **20**, 268–275 (2023).
135. Li, S. *et al.* ELI trifocal microscope: a precise system to prepare target cryo-lamellae for in situ cryo-ET study. *Nat. Methods* **20**, 276–283 (2023).

136. Jun, S. *et al.* Direct visualization of HIV-1 with correlative live-cell microscopy and cryo-electron tomography. *Structure* **19**, 1573–1581 (2011).
137. Zhang, X. *et al.* Molecular mechanisms of stress-induced reactivation in mumps virus condensates. *Cell* **186**, 1877-1894.e27 (2023).
138. Bäuerlein, F. J. B. *et al.* In Situ Architecture and Cellular Interactions of PolyQ Inclusions. *Cell* **171**, 179-187.e10 (2017).
139. Schorb, M. *et al.* New hardware and workflows for semi-automated correlative cryo-fluorescence and cryo-electron microscopy/tomography. *J. Struct. Biol.* **197**, 83–93 (2017).
140. Cai, S., Böck, D., Pilhofer, M. & Gan, L. The in situ structures of mono-, di-, and trinucleosomes in human heterochromatin. *Mol. Biol. Cell* **29**, 2450–2457 (2018).
141. Peck, A. *et al.* Montage electron tomography of vitrified specimens. *J. Struct. Biol.* **214**, 107860 (2022).
142. Chakraborty, S., Mahamid, J. & Baumeister, W. Cryoelectron Tomography Reveals Nanoscale Organization of the Cytoskeleton and Its Relation to Microtubule Curvature Inside Cells. *Structure* **28**, 991-1003.e4 (2020).
143. Fäßler, F., Zens, B., Hauschild, R. & Schur, F. K. M. 3D printed cell culture grid holders for improved cellular specimen preparation in cryo-electron microscopy. *J. Struct. Biol.* **212**, 107633 (2020).
144. Jimah, J. R. *et al.* Cryo-EM structures of membrane-bound dynamin in a post-hydrolysis state primed for membrane fission. *Dev. Cell* **59**, 1783-1793.e5 (2024).
145. Liu, J. *et al.* The palisade layer of the poxvirus core is composed of flexible A10 trimers. *Nat. Struct. Mol. Biol.* **31**, 1105–1113 (2024).
146. Mesman, R. J. A novel method for high-pressure freezing of adherent cells for frozen hydrated sectioning and CEMOVIS. *J. Struct. Biol.* **183**, 527–530 (2013).
147. Gruska, M., Medalia, O., Baumeister, W. & Leis, A. Electron tomography of vitreous sections from cultured mammalian cells. *J. Struct. Biol.* **161**, 384–392 (2008).
148. Yi, H. *et al.* Native Immunogold Labeling of Cell Surface Proteins and Viral Glycoproteins for Cryo-Electron Microscopy and Cryo-Electron Tomography Applications. *J. Histochem. Cytochem.* **63**, 780–792 (2015).
149. Creekmore, B. C., Kixmoeller, K., Black, B. E., Lee, E. B. & Chang, Y. W. Ultrastructure of human brain tissue vitrified from autopsy revealed by cryo-ET with cryo-plasma FIB milling. *Nat. Commun.* **15**, 1–12 (2024).
150. Fu, X. *et al.* AutoCLEM: An Automated Workflow for Correlative Live-Cell Fluorescence Microscopy and Cryo-Electron Tomography. *Sci. Rep.* **9**, 1–10 (2019).
151. van Driel, L. F., Valentijn, J. A., Valentijn, K. M., Koning, R. I. & Koster, A. J. Tools for correlative cryo-fluorescence microscopy and cryo-electron tomography applied to whole mitochondria in human endothelial cells. *Eur. J. Cell Biol.* **88**, 669–684 (2009).
152. Engel, L. *et al.* Lattice micropatterning for cryo-electron tomography studies of cell-cell contacts. *J. Struct. Biol.* **213**, 107791

(2021).
153. Wu, G. H. *et al.* CryoET reveals organelle phenotypes in huntington disease patient iPSC-derived and mouse primary neurons. *Nat. Commun.* **14**, 9–12 (2023).
154. Hoyer, M. J. *et al.* Combinatorial selective ER-phagy remodels the ER during neurogenesis. *Nat. Cell Biol.* **26**, 378–392 (2024).
155. Sartori, A. *et al.* Correlative microscopy: Bridging the gap between fluorescence light microscopy and cryo-electron tomography. *J. Struct. Biol.* **160**, 135–145 (2007).
156. Killilea, A. N. *et al.* Cytoskeletal organization in microtentacles. *Exp. Cell Res.* **357**, 291–298 (2017).
157. Tran, N. H. *et al.* The stress-sensing domain of activated IRE1a forms helical filaments in narrow ER membrane tubes. *Science.* **374**, 52–57 (2021).
158. Mageswaran, S. K. *et al.* Nanoscale details of mitochondrial constriction revealed by cryoelectron tomography. *Biophys. J.* **122**, 3768–3782 (2023).
159. Elad, N. *et al.* The role of integrin-linked kinase in the molecular architecture of focal adhesions. *J. Cell Sci.* **126**, 4099–4107 (2013).
160. Barad, B. A., Medina, M., Fuentes, D., Wiseman, R. L. & Grotjahn, D. A. Quantifying organellar ultrastructure in cryo-electron tomography using a surface morphometrics pipeline. *J. Cell Biol.* **222**, (2023).
161. Martins, B. *et al.* Unveiling the polarity of actin filaments by cryo-electron tomography. *Structure* **29**, 488-498.e4 (2021).
162. Eibauer, M. *et al.* Vimentin filaments integrate low-complexity domains in a complex helical structure. *Nat. Struct. Mol. Biol.* **31**, 939–949 (2024).
163. Kronenberg-Tenga, R. *et al.* A lamin A/C variant causing striated muscle disease provides insights into filament organization. *J. Cell Sci.* **134**, 1–9 (2021).
164. Lovatt, M., Leistner, C. & Frank, R. A. W. Bridging length scales from molecules to the whole organism by cryoCLEM and cryoET. *Faraday Discuss.* **240**, 114–126 (2022).
165. Leistner, C. *et al.* The in-tissue molecular architecture of β-amyloid pathology in the mammalian brain. *Nat. Commun.* **14**, 1–12 (2023).
166. Zhang, J. *et al.* VHUT-cryo-FIB, a method to fabricate frozen hydrated lamellae from tissue specimens for in situ cryo-electron tomography. *J. Struct. Biol.* **213**, 107763 (2021).
167. Wu, Y. *et al.* A practical multicellular sample preparation pipeline broadens the application of in situ cryo-electron tomography. *J. Struct. Biol.* **215**, 107971 (2023).
168. Foster, H. E., Santos, C. V. & Carter, A. P. A cryo-ET survey of microtubules and intracellular compartments in mammalian axons. *J. Cell Biol.* **221**, e202103154 (2022).
169. Atherton, J., Stouffer, M., Francis, F. & Moores, C. A. Visualising the cytoskeletal machinery in neuronal growth cones using

cryo-electron tomography. *J. Cell Sci.* **135**, (2022).
170. Ma, D., Deng, B., Sun, C., McComb, D. W. & Gu, C. The Mechanical Microenvironment Regulates Axon Diameters Visualized by Cryo-Electron Tomography. *Cells* **11**, 1–20 (2022).
171. Nedozralova, H. *et al.* In situ cryo-electron tomography reveals local cellular machineries for axon branch development. *J. Cell Biol.* **221**, (2022).
172. Guo, Q. *et al.* In Situ Structure of Neuronal C9orf72 Poly-GA Aggregates Reveals Proteasome Recruitment. *Cell* **172**, 696-705.e12 (2018).
173. Li, X. *et al.* Presynaptic Endosomal Cathepsin D Regulates the Biogenesis of GABAergic Synaptic Vesicles. *Cell Rep.* **28**, 1015-1028.e5 (2019).
174. Liu, Y. T. *et al.* Biphasic exocytosis of herpesvirus from hippocampal neurons and mechanistic implication to membrane fusion. *Cell Discov.* **6**, 1–12 (2020).
175. Fenton, A. R. *et al.* FMRP regulates MFF translation to locally direct mitochondrial fission in neurons. *Nat. Cell Biol.* **26**, 2061–2074 (2024).
176. Lučić, V. *et al.* Multiscale imaging of neurons grown in culture: From light microscopy to cryo-electron tomography. *J. Struct. Biol.* **160**, 146–156 (2007).
177. Riemenschneider, H. *et al.* Gel-like inclusions of C-terminal fragments of TDP-43 sequester stalled proteasomes in neurons. *EMBO Rep.* **23**, 1–12 (2022).
178. Aramaki, S., Mayanagi, K., Jin, M., Aoyama, K. & Yasunaga, T. Filopodia formation by crosslinking of F-actin with fascin in two different binding manners. *Cytoskeleton* **73**, 365–374 (2016).
179. Singh, D. *et al.* The molecular architecture of the nuclear basket. *Cell* **187**, 5267-5281.e13 (2024).
180. Henderson, G. P., Gan, L. & Jensen, G. J. 3-D Ultrastructure of O. tauri: Electron Cryotomography of an Entire Eukaryotic Cell. *PLoS One* **2**, e749 (2012).
181. Cai, S., Song, Y., Chen, C., Shi, J. & Gan, L. Natural chromatin is heterogeneous and self-associates in vitro. *Mol. Biol. Cell* **29**, 1652–1663 (2018).
182. Li, X. *et al.* Symmetrical organization of proteins under docked synaptic vesicles. *FEBS Lett.* **593**, 144–153 (2019).
183. Adamoski, D. *et al.* Molecular mechanism of glutaminase activation through filamentation and the role of filaments in mitophagy protection. *Nat. Struct. Mol. Biol.* **30**, 1902–1912 (2023).
184. Shimakawa, G. *et al.* Diatom pyrenoids are encased in a protein shell that enables efficient CO2 fixation. *Cell* **187**, 5919-5934.e19 (2024).
185. He, J. *et al.* Cryo-FIB specimen preparation for use in a cartridge-type cryo-TEM. *J. Struct. Biol.* **199**, 114–119 (2017).
186. Kudryashev, M. *et al.* Positioning of large organelles by a membrane- associated cytoskeleton in Plasmodium sporozoites. *Cell. Microbiol.* **12**, 362–371 (2010).

187. Kudryashev, M., Lepper, S., Baumeister, W., Cyrklaff, M. & Frischknecht, F. Geometric constrains for detecting short actin filaments by cryogenic electron tomography. *PMC Biophys.* **3**, 1–14 (2010).
188. Kudryashev, M. *et al.* Structural basis for chirality and directional motility of Plasmodium sporozoites. *Cell. Microbiol.* **14**, 1757–1768 (2012).
189. Hanssen, E. *et al.* Electron tomography of Plasmodium falciparum merozoites reveals core cellular events that underpin erythrocyte invasion. *Cell. Microbiol.* **15**, 1457–1472 (2013).
190. Walker, J. M., Marzec, B., Ozaki, N., Clare, D. & Nudelman, F. Morphological development of Pleurochrysis carterae coccoliths examined by cryo-electron tomography. *J. Struct. Biol.* **210**, 107476 (2020).
191. Marzec, B. *et al.* Three-dimensional architecture and surface functionality of coccolith base plates. *J. Struct. Biol.* **208**, 127–136 (2019).
192. Dow, L. P. *et al.* Morphological control enables nanometer-scale dissection of cell-cell signaling complexes. *Nat. Commun.* **13**, 1–12 (2022).
193. Tillu, V. A. *et al.* Precision in situ cryogenic correlative light and electron microscopy of optogenetically positioned organelles. *J. Cell Sci.* **137**, 1–16 (2024).
194. Fukuda, Y. & Nagayama, K. Zernike phase contrast cryo-electron tomography of whole mounted frozen cells. *J. Struct. Biol.* **177**, 484–489 (2012).
195. Hsieh, C. E., Leith, A. D., Mannella, C. A., Frank, J. & Marko, M. Towards high-resolution three-dimensional imaging of native mammalian tissue: Electron tomography of frozen-hydrated rat liver sections. *J. Struct. Biol.* **153**, 1–13 (2006).
196. Carter, S. D. *et al.* Distinguishing signal from autofluorescence in cryogenic correlated light and electron microscopy of mammalian cells. *J. Struct. Biol.* **201**, 15–25 (2018).
197. Gilliam, J. C. *et al.* Three-dimensional architecture of the rod sensory cilium and its disruption in retinal neurodegeneration. *Cell* **151**, 1029–1041 (2012).
198. Wu, G. H. *et al.* Multi-scale 3D Cryo-Correlative Microscopy for Vitrified Cells. *Structure* **28**, 1231–1237 (2020).
199. Hoffmann, P. C. *et al.* Tricalbins Contribute to Cellular Lipid Flux and Form Curved ER-PM Contacts that Are Bridged by Rod-Shaped Structures. *Dev. Cell* **51**, 488-502.e8 (2019).
200. Ng, C. T. *et al.* Electron cryotomography analysis of Dam1C/DASH at the kinetochore-spindle interface in situ. *J. Cell Biol.* **218**, 455–473 (2019).
201. Akey, C. W. *et al.* Comprehensive structure and functional adaptations of the yeast nuclear pore complex. *Cell* **185**, 361-378.e25 (2022).
202. Bertin, A. *et al.* Three-dimensional ultrastructure of the septin filament network in Saccharomyces cerevisiae. *Mol. Biol. Cell* **23**, 423–432 (2012).
203. Wilfling, F. *et al.* A Selective Autophagy Pathway for Phase-Separated Endocytic Protein Deposits. *Mol. Cell* **80**, 764-778.e7

(2020).
204. Collado, J. *et al.* Tricalbin-Mediated Contact Sites Control ER Curvature to Maintain Plasma Membrane Integrity. *Dev. Cell* **51**, 476-487.e7 (2019).
205. Hayles, M. F. *et al.* The making of frozen-hydrated, vitreous lamellas from cells for cryo-electron microscopy. *J. Struct. Biol.* **172**, 180–190 (2010).
206. Pierson, J. *et al.* Improving the technique of vitreous cryo-sectioning for cryo-electron tomography: Electrostatic charging for section attachment and implementation of an anti-contamination glove box. *J. Struct. Biol.* **169**, 219–225 (2010).
207. Rogers, S. *et al.* Triglyceride lipolysis triggers liquid crystalline phases in lipid droplets and alters the LD proteome. *J. Cell Biol.* **221**, e202205053 (2022).
208. De Winter, D. A. M. *et al.* In-situ integrity control of frozen-hydrated, vitreous lamellas prepared by the cryo-focused ion beam-scanning electron microscope. *J. Struct. Biol.* **183**, 11–18 (2013).
209. Millen, J. I., Pierson, J., Kvam, E., Olsen, L. J. & Goldfarb, D. S. The luminal N-terminus of yeast Nvj1 is an inner nuclear membrane anchor. *Traffic* **9**, 1653–1664 (2008).
210. Studer, D., Klein, A., Iacovache, I., Gnaegi, H. & Zuber, B. A new tool based on two micromanipulators facilitates the handling of ultrathin cryosection ribbons. *J. Struct. Biol.* **185**, 125–128 (2014).
211. Hugener, J. *et al.* FilamentID reveals the composition and function of metabolic enzyme polymers during gametogenesis. *Cell* **187**, 3303-3318.e18 (2024).
212. Leung, M. R. *et al.* The multi-scale architecture of mammalian sperm flagella and implications for ciliary motility. *EMBO J.* **40**, 1–17 (2021).
213. Maitan, P. P. *et al.* Bicarbonate-Stimulated Membrane Reorganization in Stallion Spermatozoa. *Front. Cell Dev. Biol.* **9**, 1–17 (2021).
214. Höög, J. L. & Lötvall, J. Diversity of extracellular vesicles in human ejaculates revealed by cryo-electron microscopy. *J. Extracell. Vesicles* **4**, 1–11 (2015).
215. Chen, Z. *et al.* In situ cryo-electron tomography reveals the asymmetric architecture of mammalian sperm axonemes. *Nat. Struct. Mol. Biol.* **30**, 360–369 (2023).
216. Zabeo, D., Croft, J. T. & Höög, J. L. Axonemal doublet microtubules can split into two complete singlets in human sperm flagellum tips. *FEBS Lett.* **593**, 892–902 (2019).
217. Tai, L., Yin, G., Huang, X., Sun, F. & Zhu, Y. In-cell structural insight into the stability of sperm microtubule doublet. *Cell Discov.* **9**, 1–19 (2023).
218. Kühni-Boghenbor, K. *et al.* Actin-mediated plasma membrane plasticity of the intracellular parasite Theileria annulata. *Cell. Microbiol.* **14**, 1867–1879 (2012).
219. Zens, B. *et al.* Lift-out cryo-FIBSEM and cryo-ET reveal the ultrastructural landscape of extracellular matrix. *J. Cell Biol.* **223**,

e202309125 (2024).
220. Sun, S. Y. *et al.* Cryo-ET of Toxoplasma parasites gives subnanometer insight into tubulin-based structures. *Proc. Natl. Acad. Sci. U. S. A.* **119**, 1–11 (2022).
221. Segev-Zarko, L. A. *et al.* Cryo-electron tomography with mixed-scale dense neural networks reveals key steps in deployment of Toxoplasma invasion machinery. *PNAS Nexus* **1**, 1–13 (2022).
222. Sparvoli, D. *et al.* An apical membrane complex for triggering rhoptry exocytosis and invasion in Toxoplasma . *EMBO J.* **41**, 1–26 (2022).
223. Höög, J. L., Bouchet-Marquis, C., McIntosh, J. R., Hoenger, A. & Gull, K. Cryo-electron tomography and 3-D analysis of the intact flagellum in Trypanosoma brucei. *J. Struct. Biol.* **178**, 189–198 (2012).
224. Maimon, T., Elad, N., Dahan, I. & Medalia, O. The human nuclear pore complex as revealed by cryo-electron tomography. *Structure* **20**, 998–1006 (2012).
225. de Jager, L. *et al.* StableMARK-decorated microtubules in cells have expanded lattices. *J. Cell Biol.* **224**, e202206143 (2025).
226. Kirchweger, P., Mullick, D., Swain, P. P., Wolf, S. G. & Elbaum, M. Correlating cryo-super resolution radial fluctuations and dual-axis cryo-scanning transmission electron tomography to bridge the light-electron resolution gap. *J. Struct. Biol.* **215**, 107982 (2023).
227. Lansky, Z. *et al.* 3D mapping of native extracellular matrix reveals cellular responses to the microenvironment. *J. Struct. Biol. X* **1**, 100002 (2019).
